# Combined generalist and host-specific transcriptional strategies enable host generalism in the fungal pathogen *Botrytis cinerea*

**DOI:** 10.1073/pnas.2521414123

**Published:** 2026-05-19

**Authors:** Ritu Singh, Anna Jo Muhich, Cloe Tom, Jack McMillan, Karishma Srinivas, Lucca Faieta, Celine Caseys, Daniel J. Kliebenstein

**Affiliations:** ^a^Department of Plant Science, University of California, Davis, CA 95616

**Keywords:** transcriptional plasticity, necrotrophic fungal pathogenesis, eudicot hosts, entropy, transcriptome

## Abstract

How generalist pathogens infect phylogenetically diverse hosts remains a central question in plant-pathogen biology. In particular, the extent to which broad host range is enabled by genetic variation vs. transcriptional plasticity is unclear. To investigate how variation and plasticity contribute to generalism, we studied the generalist necrotrophic fungus *Botrytis cinerea* that infects more than 1,500 plant species. Using a cross-infection matrix of 72 *B. cinerea* isolates infecting 57 plant genotypes across 15 eudicot species, we identified general and host-dependent fungal components of lesion formation. Transcriptome profiling at 48 h postinoculation revealed two distinct pathogen gene modules: 1) a set of general lesion-associated genes showing similar expression across hosts but varied among isolates; and 2) a set of high-entropy, host-inducible genes, organized into distinct coregulated modules that respond dynamically to specific host cues. Both gene sets were genomically dispersed, lacking structural clustering, and were under different levels of selective constraints. Our results demonstrate that *B. cinerea* employs a modular transcriptional strategy that integrates a core metabolic program along with a plastic, host-responsive regulatory network to achieve broad host colonization. This study presents the most comprehensive cross-species cotranscriptomic dataset to date for any fungal phytopathogen, highlighting transcriptional plasticity as a key mechanism underlying generalism in plant–fungal interactions. Moreover, the identification of conserved fungal gene targets across diverse hosts offers a foundation for developing broad-spectrum resistance strategies in multiple crops.

## Significance

Pathogens that infect multiple plant species threaten global food security, but how they successfully colonize such diverse hosts remains unclear. This study uncovers how the fungal pathogen *Botrytis cinerea* leverages a modular transcriptional strategy- combining a conserved, metabolism-driven core program with flexible, host-inducible gene expression, to infect a wide range of eudicot plants. By systematically analyzing fungal gene expression across diverse eudicot host species and 72 pathogen isolates, this work provides a powerful framework to understand how pathogens generalize across plant species. These findings shift the focus from genetic content to transcriptional adaptability and reveal conserved fungal targets for designing broad-spectrum disease resistance in crops.

How and why some pathogens can infect hundreds of hosts while others are constrained to a single host remains an unfilled gap in our understanding of host–pathogen interactions and adaptability. Fungal pathogens show a wide range of infection lifestyles, and these lifestyles vary in their host-range patterns. Obligately biotrophic species often display narrow host specificity while hemibiotrophic and necrotrophic species can show broader host ranges (1). Some nonobligate biotrophic also show wide host ranges, such as *Claviceps purpurea*, which infects more than 400 grass species (2). In contrast, several necrotrophic groups include highly specialized species, including members of *Cochliobolus* and *Ascochyta* (3, 4). The occurrence of broad and restricted host ranges across all fungal lifestyles establishes a powerful comparative framework to determine why some pathogens evolve extreme specialization while others maintain compatibility with many host lineages.

A pathogen’s host range dynamic can be shaped by a complex interplay of genetic factors, environmental heterogeneity, and ecological interactions, which collectively drive host shifts, range expansions, or contractions. Furthermore, classical coevolutionary models, especially the gene-for-gene model, explain specialization through tight associations between resistance genes and pathogen effectors/virulence factors (5–9). While these models provide a mechanistic understanding of pathogen specialization, they offer less insight into how generalist pathogens overcome the diverse barriers spread across hosts to infect a diverse range of hosts.

Several genetic mechanisms have been suggested to enable pathogens’ host range expansion, including mutation, hybridization, genome rearrangement, and horizontal gene transfer (10–12). While these gradual processes are important, they do not fully explain the ability of some pathogens to rapidly shift hosts. Recent work has begun to link these broad host shifts to phenotypic plasticity, the ability of a single genotype to produce different phenotypes in response to environmental or biotic cues (13). Three conceptual models describe how plasticity may facilitate host shifts: 1) ecological fitting, where pathogens exploit conserved traits across related hosts; 2) adaptive plasticity, in which induced traits enhance fitness on novel hosts; and 3) nonadaptive plasticity, where initial maladaptive responses expose cryptic genetic variation that may promote long-term adaptation (14). While these various plasticity models have been studied in modulating abiotic stress responses (15–17), their relevance to host–pathogen interactions, where both partners dynamically influence each other’s biology, remains elusive.

Unlike viruses and bacteria, which readily adapt to hosts via high mutation rates, eukaryotic pathogens may rely more on transcriptional reprogramming to facilitate broad host colonization (18). For instance, in the ectoparasite crustacean *Tracheliastes polycolpus*, transcriptional plasticity enabled transcriptomic shifts across native and novel fish hosts (19). In the plant pathogen *Fusarium oxysporum*, infection on nonvascular liverworts involved upregulation of conserved effector genes, while lineage-specific effectors associated with angiosperm infection remained silent (20). Similarly, *Fusarium virguliforme* exhibits host-dependent transcriptional programs that differ between the pathogenic lifestyle on soybean and endophytic colonization on maize (21). Cotranscriptomic analyses of a single isolate of the generalist fungal pathogen *Sclerotinia sclerotiorum* across six host species revealed both generalist and polyspecialist transcriptional strategies, with distinct gene expression programs deployed depending on the host, suggesting that plasticity can enable a flexible generalist infection strategy (22).

While these studies highlight the potential role of transcriptional plasticity in facilitating infection on different hosts, most have focused on either single pathogen isolates or a limited set of hosts. Thus, it remains unclear how genetic diversity in the pathogen populations modulates transcriptional plasticity across phylogenetically diverse hosts. Generalist pathogens may employ overlapping strategies, including standing genetic variation, core and lineage-specific virulence programs, and transcriptional plasticity, to overcome diverse host defenses. Dissecting the relative contribution of each mechanism requires a broad, systematic, and phylogenetically informed approach that captures both host diversity and pathogen intraspecific variation.

*Botrytis cinerea* provides a powerful model to address these questions. This broad host-range necrotrophic fungus infects over 1,500 plant species across angiosperms, gymnosperms, and mosses, with a notable preponderance among eudicots (23, 24). While *B. cinerea* exhibits extensive standing genetic diversity and polygenic virulence architecture (25–29), the extent to which *B. cinerea* transcriptionally adapts to diverse hosts remains unresolved. A key advantage enabling this question is that *B. cinerea* can readily infect many host species, and its transcripts can be simultaneously measured from host–pathogen RNA-seq data, enabling efficient and scalable cotranscriptome profiling (30).

In this study, we use the *B. cinerea*–eudicot pathosystem to investigate how phenotypic and transcriptional plasticity, alongside genetic diversity of both host and pathogen, contributes to the generalist lifestyle of *B. cinerea*. We assayed lesion development across 15 eudicot host species spanning eight orders. Species within each order belonged to the same family. Four genotypes were included per species, and all hosts were infected with 72 genetically diverse *B. cinerea* isolates, capturing a wide range of natural variation (30–35). To quantify transcriptional dynamics during infection, a cotranscriptome dataset was generated for 10 representative host species that produced reliable *B. cinerea* reads. These species represent six eudicot orders and were infected with the same panel of 72 isolates. This large-scale, phylogenetically informed design enables us to explore how host evolutionary distance and pathogen genetic diversity shape infection outcomes and fungal gene expression programs. By integrating phenotypic and transcriptomic data across diverse host–pathogen combinations, we aim to uncover how plasticity and genetic diversity drive broad host colonization.

## Results

### Comparative Lesion Development.

To investigate the interaction of a *B. cinerea* population across diverse host species, we infected 57 plant genotypes representing 15 eudicot species from eight taxonomic orders with 72 *B. cinerea* isolates along with mock (Dataset S3). This design created a total of 4,161 host by fungal isolate infection combinations at 72 h postinoculation (hpi) (*SI Appendix*, Fig. S1 and Datasets S1 and S2). Notably, lesions typically became visible only after 48 hpi in most species, preventing digital image analysis of earlier time points. To understand how the lesions further progressed from 72 to 96 hpi, we calculated the relative growth rate [RGR = (lesion 96 hpi – lesion 72 hpi)/ 24] of each isolate. The RGR showed a linear relationship with lesion size at 72 hpi across all species, suggesting that lesion size at 72 hpi is a reliable proxy for *B. cinerea* growth potential. Notably, the slope of the relationship varies among species (*SI Appendix*, Fig. S2*A*). Parsley, Pepper, Cowpea, Bean, and Cucumber exhibited faster growth from 72 to 96 hpi, whereas Chard and Celery showed slower progression, likely reflecting differences in postpenetration host responses (*SI Appendix*, Fig. S2). The rank order of the isolate virulence at 72 hpi vs. 96 hpi did not change within a host, as expected for a linear relationship (*SI Appendix*, Fig. S2*B*). Therefore, the lesion area at 72 hpi was selected as the optimal time point for investigating host–pathogen interaction dynamics while avoiding any potential constraints on lesion development caused by tissue availability.

### Quantitative Variation in Host Susceptibility to *B. Cinerea* across Eudicot Plant Species.

To assess the contribution of host and pathogen genetic diversity to variation in lesion size, we used a linear mixed model within individual plant species that accounted for experimental design variables. These experimental factors explained a significant proportion of variance in nearly all species (*SI Appendix*, Fig. S3*A*). Within each individual host species, genetic variation among the 72 *B. cinerea* isolates explained the largest proportion of lesion size variance, ranging from 15% to 45% of the total (*SI Appendix*, Fig. S3*A*). The interaction between host genotype and isolate contributed an additional 5% to 11% of the total variance, while host genotype alone explained a smaller but statistically significant fraction (0.6% to 6%). This indicates that within species, pathogen genetic diversity is a primary driver of lesion size variation, with host genotype playing a smaller but significant role.

Comparing lesion area across the 15 host species showed substantial interspecific variation. Celery exhibited the smallest lesions, indicating high tolerance to *B. cinerea*, whereas Sunflower developed the largest, reflecting high susceptibility (*SI Appendix*, Fig. S3*C* and Datasets S1 and S2). Genotypes within each plant species showed a similar range of lesion sizes (*SI Appendix*, Fig. S7). At a broader phylogenetic level, many species from the Rosids clade, such as *Arabidopsis*, Mustard, and Cowpea, displayed lower susceptibility than several Asterids, such as Lettuce and Sunflower. Species from the Caryophyllales (Spinach and Chard) were among the resistant species. These patterns highlight the diversity of the disease outcome and suggest a phylogenetic signal in *B. cinerea–*eudicot interactions across eudicots.

### Host–Pathogen Interaction Patterns are Predominantly Shaped at the Species Level.

**Fig. 1.**
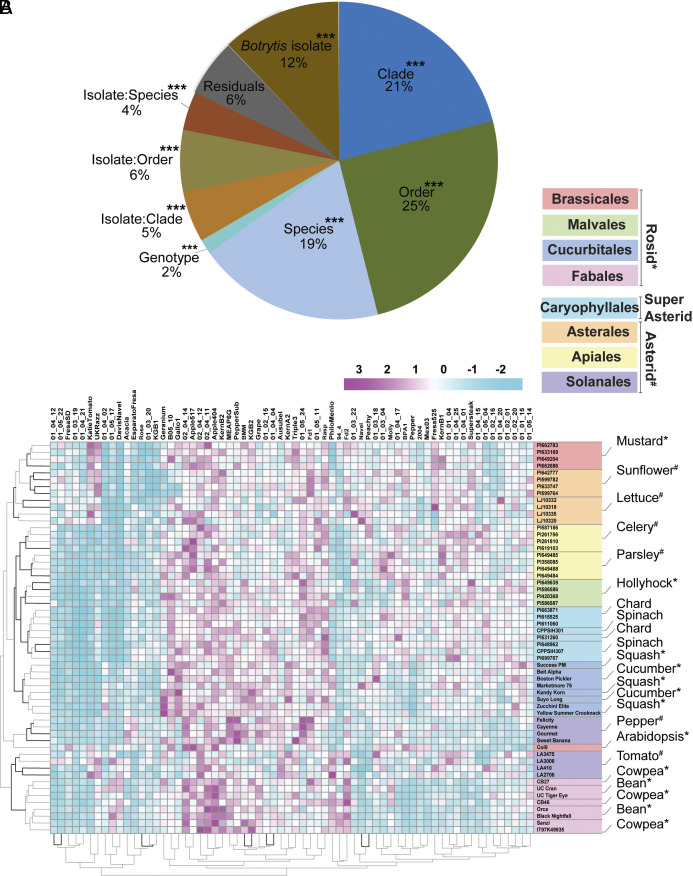
Plant susceptibility to *B. cinerea* shows species and order-specific patterns across eudicot phylogeny. (*A*) Variance partitioning from a multihost linear model using LS means derived from log transformed lesion area data. The pie chart shows the percentage of total variance in lesion area explained by host phylogenetic levels (clade, order, species, and genotype), *B. cinerea* isolate, and their interactions. Host taxonomic levels are nested (Clade > Order > Species > Genotype). For visual clarity, nested terms are simplified: Genotype represents Clade:Order:Species:Genotype, and Species represents Clade:Order:Species. Interaction terms follow a similar abbreviated convention (e.g., Isolate:Species). This model quantifies both host and pathogen contributions to observed lesion variation across the full dataset. Asterisks (***P < 0.001) indicate the statistical significance of each variance component. Comparative variance partitioning using raw lesion data and random effect subtraction-based methods are available in *SI Appendix*, Fig. S6 *C* and *D*, respectively. (*B*) Heatmap showing standardized (z-scored) least squares mean lesion areas from infection assays between 72 *B. cinerea* isolates (columns) and 57 plant genotypes (rows). Each row corresponds to a unique plant genotype, with genotype names followed by their respective species. All plant species are represented by four genotypes, except Arabidopsis, which has a single genotype (Col-0). Colored sidebars indicate plant taxonomic orders; species labeled with * belong to the Rosid clade, **#** denotes Asterid, and unlabeled species belong to the Super-Asterid group. Lesion values were Z-score standardized (centered and scaled) per column (per isolate) to account for variability in isolate virulence. Hierarchical clustering was performed using Euclidean distance and the complete agglomerative linkage method. The heatmap was generated using the pheatmap package in R. Dendrograms indicate hierarchical clustering of isolates and host genotypes; cluster stability was assessed via multiscale bootstrap resampling (n = 1,000), with bold branches denoting clusters with ≥95% bootstrap support.

To evaluate whether host-specific differences in lesion size reflect the hosts’ evolutionary relationships, we assessed how eudicot phylogeny is associated with *B. cinerea* lesion formation. A multihost nested linear mixed model across the host phylogeny showed that the largest contribution to the percentage of variance in lesion area is explained by the orders, followed sequentially by clade, species, and genotype (Fig. 1*A*). When considering interaction with the pathogen, isolate-by-species, isolate-by-order, and isolate-by-clade effects were all significant, but each explained substantially less variation than the corresponding host phylogenetic levels. *B. cinerea* isolates main effect yet contributed less variation than higher level host structure in the multihost model. The order of effects remained similar when models were fitted using log transformed lesion values, raw lesion values in a two stage framework, and a random effect subtraction approach (Fig. 1*A* and *SI Appendix*, Fig. S6 *C* and *D*). Thus, the order of phylogenetic effects was robust to modeling strategy. These findings suggest that while *B. cinerea* isolate variation is a major driver of lesion differences within individual species, across the host phylogeny, the diversity of disease outcome is primarily shaped by hosts followed by their interactions with the pathogen.

To further explore this phylogenetic structure, we performed hierarchical clustering of lesion area across all host genotypes and *B. cinerea* isolates. Most host genotypes clustered within their respective species, with notable exceptions in Bean, Cowpea, and one Chard genotype, where genotypes did not cluster together within the species but within the order (Fig. 1*B*). Order-level grouping was also evident for most species, supporting a potential phylogenetic signal in lesion formation. However, the species and order level clustering collapsed at the clade level with Rosids and Asterids being interspersed (Fig. 1*B*). While deeper taxonomic structures were statistically significant, their impact on disease outcome is limited compared to lineage-specific variation. These patterns suggest that *B. cinerea*–eudicot interactions are largely influenced by variation within or between closely related host species or rather than by deeper evolutionary branches. The current sampling includes only one family per order, which constrains the phylogenetic resolution but was required for experimental feasibility. Broader sampling across families and additional orders will be required to test how lesion outcomes scale across a broader eudicot sample.

### General and Host-Dependent Lesion-Forming Potential in *B. Cinerea*.

The modest but statistically significant isolate main effect across the phylogenetically diverse hosts suggests a host-independent virulence component among the *B. cinerea* isolates. To estimate such effects, we calculated the average lesion size of each isolate across all hosts. This metric, hereafter called general lesion, captures the lesion-forming potential of an isolate across all hosts. Additionally, we generated a variable called host-dependent lesion, which was calculated as the mean lesion area produced by each isolate within a single host species (*Materials and Methods*) (29, 35).

To assess the relationship between general and host-dependent lesion potential, we performed Pearson correlation analyses. Across all host species, we observed significant positive correlations, indicating that isolates with higher overall mean lesions (general lesion) also formed larger lesions within each specific host species (host-dependent lesion) (*SI Appendix*, Figs. S8*A* and S9). The strength of this correlation varied widely among host species and orders. For example, in Caryophyllales (Spinach and Chard), general and host-dependent lesions were highly correlated (r = 0.79 and 0.81, respectively), indicating that disease outcomes in these hosts are driven by a combination of general lesion potential and some host-specific properties. In contrast, Fabales (Bean and Cowpea) showed weaker correlations (r = 0.34 and 0.34, respectively), implying a greater contribution of host-specific interactions in shaping disease outcomes. These results indicate that while general aggressiveness partly contributes to lesion formation across all hosts, the disease outcome is heavily influenced by the host-specific aspects of the interaction.

To capture deviations from the general lesion trend, we first calculated the residuals from the relationship between an isolate’s average lesion size across all hosts (general lesion, leave one out approach) and its specific lesion on each individual host (36). These raw residuals capture specific isolate-by-host deviations that are not explained by the overall aggressiveness of the isolate; a positive residual indicates that an isolate forms a larger lesion on a specific host than general aggressiveness, while a negative residual indicates lower than general aggressiveness. To derive a single metric of deviation for each isolate, we calculated the residual standard error (σ) from the regression of host-dependent lesions on general lesion across species. This approach accounts for the sum of squared residuals and adjusts for degrees of freedom based on the number of host species sampled for each isolate, providing a statistically robust measure of host-range variance. Comparing the general lesion values to these residual standard error (σ) scores show a statistically significant but modest relationship (*R^2^* of 0.21 and *P* < 0.001; *SI Appendix*, Fig. S8*B*). This indicates that an isolate’s general aggressiveness across all host species is largely independent of its host-specific lesion formation capacity.

To directly test for host specialization, we assessed whether the seven isolates collected from hosts tested in this assay [Brassica (*Ausubel*), Tomato (*Katie Tomato*, *KGB1*, *KGB2*, *Supersteak*, and *Triple3*), and Pepper (*Pepper*)] were more virulent on their respective source host than other hosts. Across all hosts, none of these seven isolates prefer their respective source host; instead, they form bigger lesions on unrelated hosts (Datasets S1 and S2 and *SI Appendix*, Fig. S10). These results suggest limited evidence for strict host specialization among our collection of isolates, aligning with previous studies (34, 37–40).

### Early *B. Cinerea* Transcript Abundance Partially Predicts Lesion Development in a Host-Dependent Manner.

The ability of nearly all the *B. cinerea* isolates to infect all the tested host plants suggests that each isolate likely can adjust to each host. As such, this indicates that *B. cinerea* may plastically modulate its transcriptome to enable lesion formation on the different hosts. To test this, we quantified how fungal gene expression changes across the host species, pathogen isolates, and their interaction. We conducted a cotranscriptome analysis at 48 hpi across ten eudicot species infected with 72 *B. cinerea* isolates (*SI Appendix*, Fig. S1 and Dataset S3). As an initial analysis, we tested if early-stage fungal transcript abundance could serve as a predictive marker for subsequent lesion development. For this, we modeled the relationship between the total amount of *B. cinerea* transcripts at 48 hpi with the resulting lesion formed at 72 hpi across eudicot hosts using linear regression. We postulated that the proportion of fungal-mapped reads relative to total mapped reads may be a proxy for fungal biomass (30, 41). For most species, early fungal transcript abundance showed a moderate positive correlation with both host-dependent lesion and general lesion ability, with maximum *r* values around 0.69 (*SI Appendix*, Figs. S8*A*, S11, and S12). Hosts such as Sunflower, Lettuce, and Bean showed nonsignificant correlations for host-dependent lesion but significant modest correlation for general lesions. These results suggest that early fungal gene expression partially connects with subsequent lesion formation in a host-dependent manner as measured by lesion size. Furthermore, as *B. cinerea* colonizes internal plant tissues in three dimensions with genetic × host variation in hyphal density per unit volume, measuring surface lesion area is focusing on the tissue lost by the plant (35, 42).

### Genetic Variation in the Pathogen and Between Hosts Alter *B. Cinerea* Transcriptome.

**Fig. 2.**
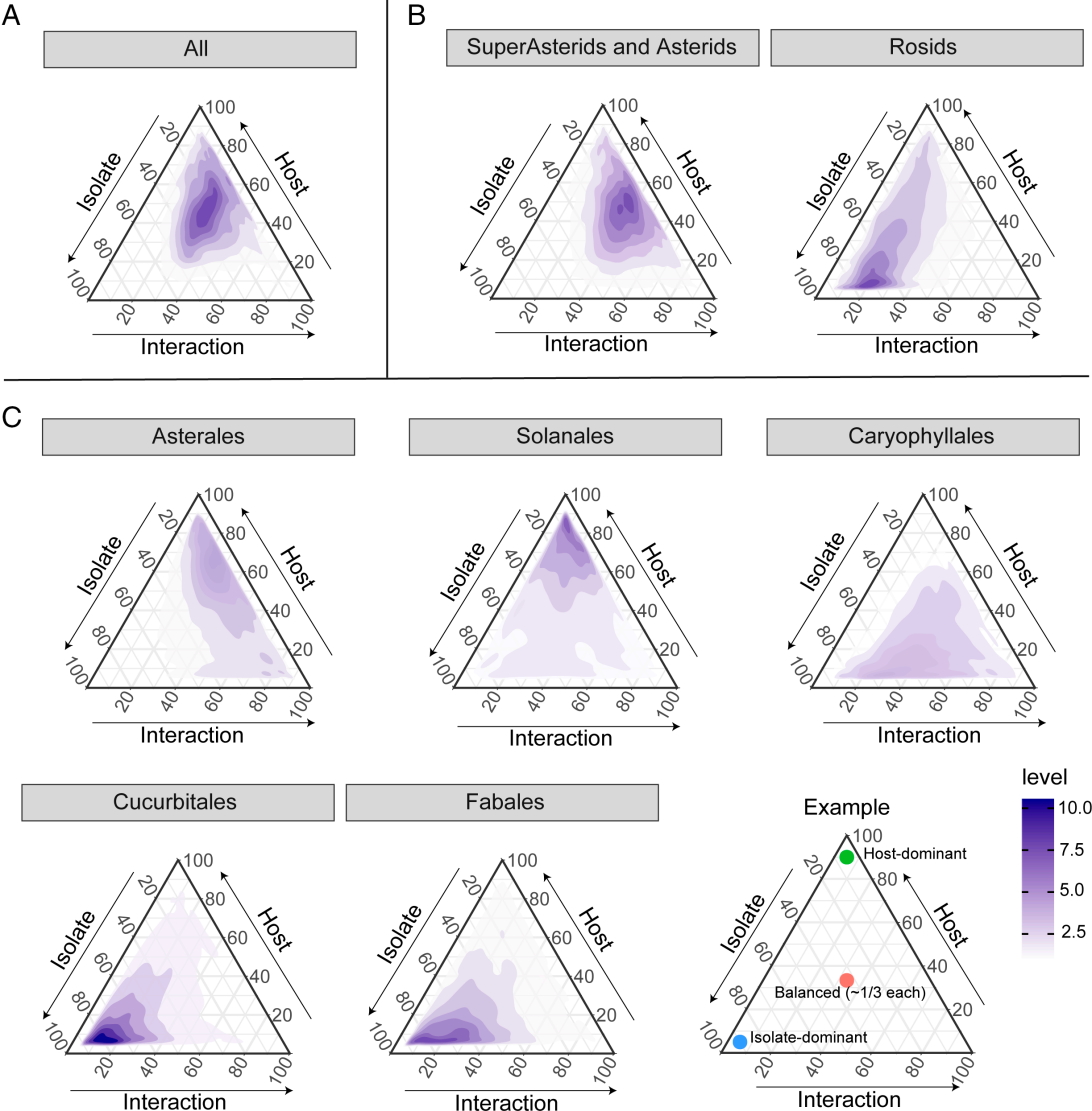
Differential genetic contribution of host and pathogen isolates to *B. cinerea* transcript variation across the full 10-host eudicot panel. Ternary plots illustrate the proportion of total heritability in *B. cinerea* gene expression explained by host plant species, *B. cinerea* isolate, and their interaction. Models were fitted separately at multiple phylogenetic levels: (*A*) across all host species (All), (*B*) within clades, and (*C*) within orders. The panel labeled Super-Asterids and Asterids integrates data from Asterales, Solanales, and Caryophyllales. Color contours (level) represent relative gene density. An annotated example ternary plot is provided to illustrate how to interpret component contributions.

To disentangle the contributions of host, pathogen, and their interaction to *B. cinerea* transcripts expression, we fit linear models for each gene across all host–pathogen combinations within a single model and estimated broad-sense heritability (*H^2^*) for each genetically variable component (28, 34). Across the full dataset, 11,415 fungal genes were expressed, of which 6,824, 1,826, and 2,768 genes were significantly influenced by genetic variation in the host, pathogen, and host–pathogen interaction, respectively (Dataset S4). These indicate that across the full dataset, the fungal transcripts were primarily influenced by the host and host–pathogen interactions rather than the pathogen alone (Fig. 2*A*; average *H^2^*: host = 0.14, pathogen = 0.07, interaction = 0.10).

To ascertain if these relative contributions vary across evolutionary lineages, we performed analyses at the clade and order levels (Dataset S4 and Fig. 2 *B* and *C*). The patterns differed across groups: in Asterales, fungal gene expression was largely shaped by the host and interaction; in Solanales, all three components contributed evenly; and in Fabales and Cucurbitales, variation was primarily driven by the pathogen. Caryophyllales showed a unique pattern, with high heritability explained by both pathogen and host–pathogen interactions, but relatively less from the host alone. These together suggest that *B. cinerea* transcriptional responses genetically differ across isolates and are highly plastic to host species variation.

### Low-Entropy Genes Represent a Stable, Nonvirulence Core Transcriptome.

**Fig. 3.**
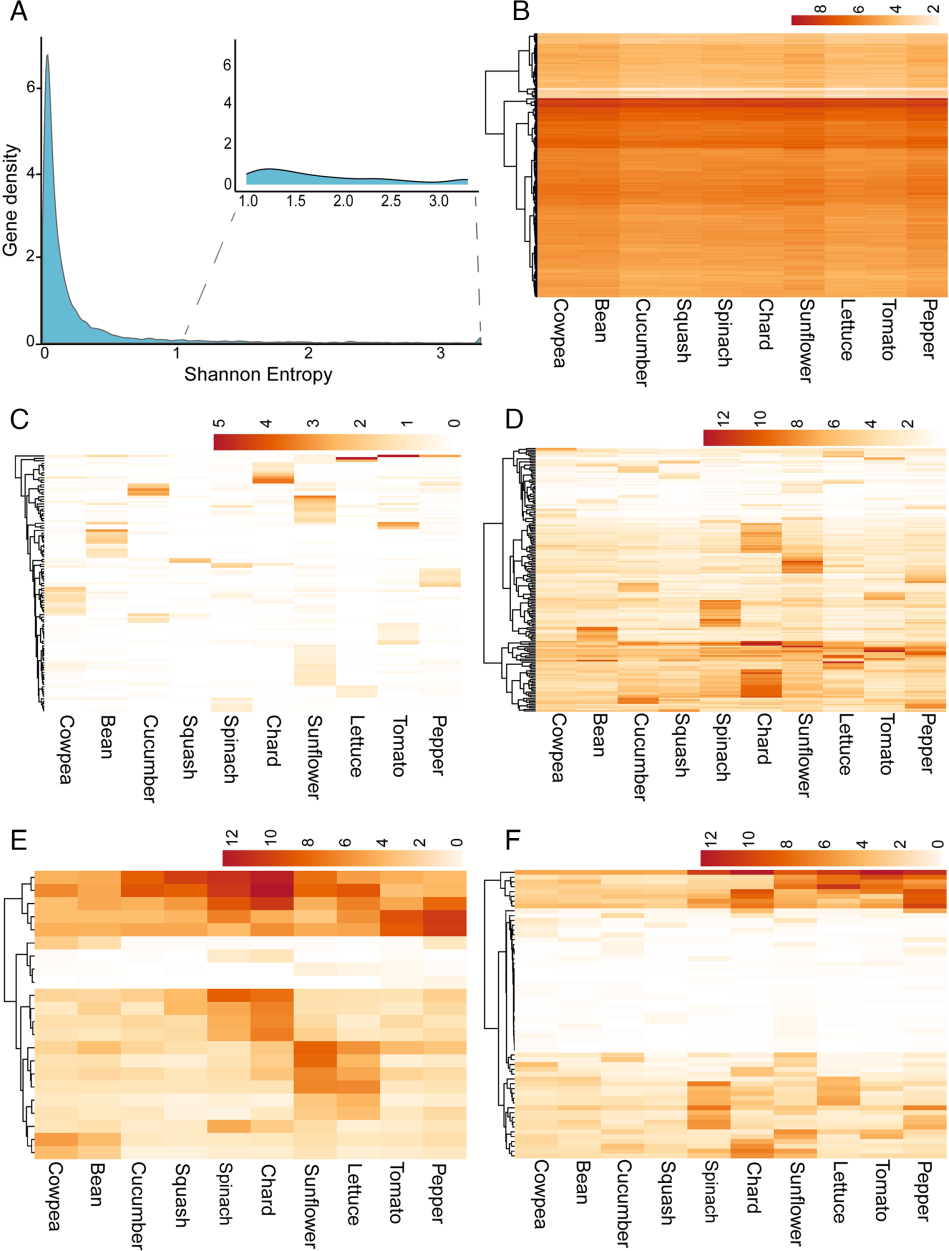
Conserved and host-specific *B. cinerea* gene expression across 10 eudicot hosts. (*A*) Density plot of Shannon entropy across *B. cinerea* genes calculated from host-averaged normalized counts per million (CPM) expression values. The *Inset* zooms into the *Right* tail of the distribution, highlighting high-entropy genes. (*B*–*F*) Heatmaps showing log^2^ (CPM + 1) expression values for each of 10 host species, averaged across 72 *B. cinerea* isolates. In all panels, normalized CPM values were transformed to log^2^ (CPM + 1) for visualization only, to improve dynamic range while preserving relative expression patterns. No Z-score scaling was applied to preserve absolute expression differences; the color scale represents increasing expression intensity from low (light) to high (dark). Rows (genes) were hierarchically clustered based on expression similarity (Euclidean distance, complete linkage), while columns (hosts) are ordered according to plant phylogeny. A full isolate-level expression profile is provided in *SI Appendix*, Figs. S13*A*, S20 *B* and *C*, and S21. (*B*) Low entropy genes (lowest 500 genes): Genes with consistent expression across most hosts, indicative of broadly conserved transcriptional activity. (*C* and *D*) High-entropy genes with elevated expression in a single host (single-host-specific): A gene was defined as specific to a single host if its expression in that host was ≥1 standard deviation (SD) above its mean expression across all hosts. (*C*) 262 genes expressed in only a few hosts, with strong elevated expression (≥1 SD) in a single host. (*D*) 172 genes expressed across all hosts, but with expression ≥1 SD higher in one host compared to others. (*E* and *F*) High entropy genes (specific to two or three hosts): Genes showing higher expression (≥1 SD above their mean expression across all hosts) in two or three hosts, compared to the remaining hosts, representing moderately specific expression. (*E*) 22 genes with high expression in phylogenetically related hosts, suggesting order-specific regulatory responses. (*F*) 60 genes are highly expressed in two or three phylogenetically unrelated hosts.

The above analysis suggests that *B. cinerea* genes have diverse expression patterns across host plants. To better classify how the pathogen genes respond across hosts, we calculated the Shannon entropy for each gene using the average normalized counts per million (CPM) across all isolates for each host species (Fig. 3*A*). Entropy was computed directly from raw normalized CPM proportions without further transformation, as the metric is defined on relative abundance distributions and is suggested by published best practices (43). Low-entropy genes are uniformly expressed across all hosts, while high-entropy genes exhibit host-specific expression patterns. Using the Jenks natural breaks algorithm, genes were classified into three entropy categories: low (n = 1), intermediate (n = 10,828), and high (n = 589) (Dataset S5).

Since only one gene was assigned to a low-entropy class, we selected 500 genes with the lowest entropy values (≤0.015) for further analysis (Dataset S6). While these low-entropy genes are influenced by variation in host and isolate, their expression patterns are largely consistent across all samples (Fig. 3*B* and *SI Appendix*, Figs. S13*A* and S14). Given their relatively stable expression pattern, we did not expect strong coexpression patterns among these genes. Consistent with this, coexpression network analysis revealed that only 38 out of 500 low-entropy genes (8%) grouped into a single module across the full isolate-host dataset (*SI Appendix*, Fig. S13*B*). Functional enrichment analysis of all low-entropy genes showed significant overrepresentation of kinase activity, DNA and protein binding, and core biosynthetic and regulatory processes (*SI Appendix*, Fig. S13 *C* and *D*). Comparing these gene expression profiles in planta and in vitro on artificial media showed that all 500 genes were expressed under both conditions, with 90% showing a similar expression level between in planta and in vitro (*SI Appendix*, Fig. S15*A*). Correlating these gene expression with host-dependent lesion estimates identified no significant correlations (Dataset S7). As such, we hypothesize that these low-entropy genes represent a core expression program for *B. cinerea,* independent of the environment or plant.

### General Lesion-Associated Genes Are Enriched in Peroxisomal and Core Metabolic Processes.

**Fig. 4.**
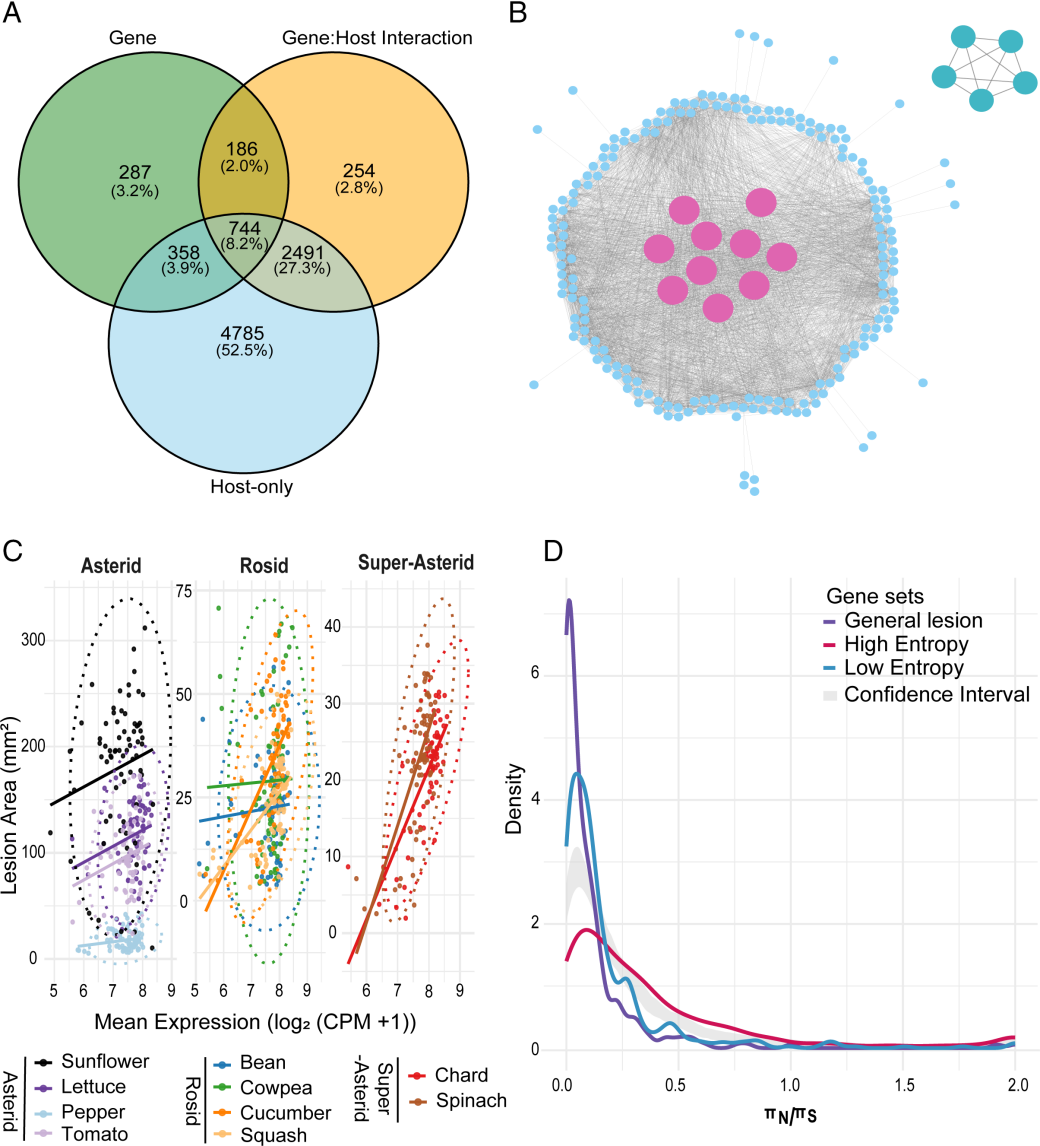
General lesion-associated module in *B. cinerea* and evolutionary constraint across general, host-specific, and constitutive gene sets. (*A*) Venn diagram showing the number of genes significantly associated with general lesion size (Gene), host identity (Host-only), and gene-by-host interaction (Gene:Host Interaction) based on individual gene linear models. A total of 287 genes were uniquely associated with the general lesion term and not confounded by host or interaction effects; these were retained as general lesion-associated candidates for further analysis. (*B*) Coexpression network of general lesion-associated genes constructed using absolute Pearson correlation (|R| ≥ 0.7) across all isolates and hosts. Nodes represent genes, and edges represent significant coexpression relationships. Pink nodes indicate hub genes within the main module. A second smaller module in the *Top* *Right* comprises five *Botcinic acid* (*BOA*; *Bcboa1*, *Bcboa2*, *Bcboa4*, *Bcboa6*, *Bcboa9*) genes. (*C*) Scatterplots showing the relationship between mean expression of general lesion-associated genes [log^2^ (CPM +1)] and host-dependent lesion area (mm^2^) across isolates for each host species. Hosts are grouped by phylogenetic clade (Asterid, Rosid, Super Asterid), and colored lines represent linear regressions for each species. Ellipses denote 95% confidence intervals for each host group. Each point represents an isolate. Rho and *P*-values for host-wise Spearman correlations are provided in Dataset S7. (*D*) Density distribution of nonsynonymous to synonymous nucleotide diversity ratios (πN/πS) for three *B. cinerea* gene sets: general lesion-associated, host-specific high-entropy, and constitutive low-entropy genes. The shaded gray area represents the 95% confidence interval of 100 permutations comprising 500 random genes.

The majority (88%) of *B. cinerea* genes fell into the intermediate Shannon entropy category, indicative of broadly distributed but variable expression patterns across isolates and hosts (Dataset S5). We hypothesized that this category may include candidates contributing to the variation in isolates’ general lesion-forming potential across hosts. To test this, we performed a linear model across all genes regardless of entropy and 72 *B. cinerea* isolates, correlating general lesion to fungal gene expression while controlling for host species and gene-by-host interaction effects. This approach identified 287 genes significantly associated with general lesion variation across all hosts and no host dependency (Fig. 4*A* and Dataset S8). As expected, almost all these genes belonged to the intermediate entropy category, with only two genes being identified from within the high-entropy genes. These general lesion-associated genes showed similar expressions across all hosts but varied among isolates (*SI Appendix*, Fig. S16).

To determine whether these genes were plant-induced or more broadly expressed, we compared their expression in vitro and in planta conditions. All 287 genes were expressed in both conditions; however, 39% exhibited elevated expression in planta, 45% showed similar expression in both conditions, and 16% showed higher expression in vitro (*SI Appendix*, Fig. S15*B*). These results indicate that this gene module exhibits conditional plasticity, with many genes varying in expression depending on the growth environment. We next assessed the extent to which expression of these general lesion–associated genes were correlated with host-dependent lesion size. Significant positive correlations were observed in Chard (ρ = 0.69), Cucumber (ρ = 0.58), Spinach (ρ = 0.58), Squash (ρ = 0.48), Cowpea (ρ = 0.23), and Lettuce (ρ = 0.24), while the remaining four hosts showed no significant associations (Dataset S7 and Fig. 4*C*).

To identify potential mechanistic networks, we developed coexpression networks using these 287 genes expression across all the hosts. This identified two coexpression networks that differ by functional enrichment analysis. The large module, including 207 genes, was enriched for peroxisomal transport, fatty acid catabolism, and other core metabolic processes, while the smaller module contained five *Botcinic acid* (*BOA*) biosynthetic genes (*Bcboa1, Bcboa2, Bcboa4, Bcboa6, and Bcboa9*) (Fig. 4*B* and *SI Appendix*, Fig. S17). Fatty acid catabolism is largely occurring in the peroxisome which suggests a potential coordinated expression of genes involved in organelle function, lipid breakdown, and basic cellular metabolism, along with a selected phytotoxin pathway underlies variation in general lesion formation across isolates. However, it is also possible that the elevated expression of these metabolic functions could be a physiological response to the increased energetic demands of faster growth rates likely occurring in high-virulence isolates, rather than a direct upstream driver of virulence itself. Future functional studies are required to distinguish between these causal and consequential roles.

Interestingly, several canonical virulence factors, including cell wall-degrading enzymes (e.g., *PG1*), phytotoxins from the *Botrydial* (*BOT*) cluster, and other *BOA* cluster genes, were not identified among the general lesion-associated gene set. This is notable given previous hypotheses that such genes should be uniformly expressed across host species (27, 44, 45). Independent examination of these canonical genes’ expression profiles revealed that most of them were within the intermediate entropy category, but with moderate host-dependent variation in expression (*SI Appendix*, Figs. S18 and S19). This suggests that these genes expression are plastic in a host-dependent manner, making their importance for virulence variable across all hosts and may reflect alternative fungal strategies or host-specific resistance mechanisms.

### Host-Specific Transcriptional Expression Patterns.

Next, to identify host-specific transcriptional responses, we analyzed 589 high-entropy genes that showed substantial expression variability across hosts. Using z-score normalization of log^2^ (CPM + 1) values, we defined a gene as specific to a single host if its expression in that host was at least one standard deviation (SD) above its mean expression across all hosts. Based on this criterion, we identified 434 genes as single host-specific, which clustered into two distinct expression patterns based on their background expression across all hosts: i) 262 genes were largely unexpressed except within the single host (Fig. 3*C* and *SI Appendix*, Fig. S20*B*); and ii) 172 genes had a low background expression across all hosts with elevated expression in a single host (Fig. 3*D* and *SI Appendix*, Fig. S20*C*). Supporting the host-specific in planta role for these 434 single host-specific genes, 337 of these genes are expressed only in planta with no expression in vitro. Of the 97 genes also expressed in vitro, 67% had higher expression in planta (*SI Appendix*, Fig. S15*C*). Together, this indicates that these host-specific genes are mainly functional during colonization on specific hosts. The number of host-specific *B. cinerea* genes was uneven across species, ranging from 83 genes specific to Sunflower to 18 genes specific to Squash (*SI Appendix*, Fig. S20*A* and Dataset S9), suggesting that the degree of host-specific fungal genes varies substantially among hosts.

In addition to these single host-specific genes, we also identified 82 high-entropy genes with elevated expression in two or three hosts (Fig. 3 *E* and *F*, *SI Appendix*, Fig. S22, and Dataset S10). Among these, 22 showed clustering with host order: Asterales (n = 7), Caryophyllales (n = 9), Fabales (n = 3), and Solanales (n = 3), suggesting limited phylogenetic coherence in host-specific transcriptional responses (Fig. 3*E*).

Investigating the role of genetic variation in influencing host-specific genes expression variation showed that they were predominantly shaped by the host. There was some contribution from isolate variation, but solely through host–pathogen interactions (*SI Appendix*, Fig. S14). While the host environment was the primary driver, we observed variation in the breadth of the response across the isolate population that corresponded to the two gene groups identified above. For the 262 genes that were largely unexpressed outside the specific host, a few genes had higher expression (exceeding one SD) across nearly all isolates, but the majority of the genes showed significant variation across the isolate population (average of 34% of isolates exceeded the threshold). In contrast, the second group of 172 genes that were highest expressed in one host while showing lower expression in the other hosts showed a more conserved response, with an average of 78% isolates exceeding the threshold (Fig. 3 *C* and *D* and *SI Appendix*, Figs. S20 *B* and *C* and S21). This confirms that while a single host consistently induces higher expression relative to other hosts (Fig. 3 *C* and *D*), there is population-level diversity of this plasticity. Notably, as seen in *SI Appendix*, Fig. S21, the fraction of isolates consistently inducing these host-specific genes varies significantly by host susceptibility. In highly susceptible species such as Sunflower, Lettuce, and Tomato, which are readily colonized by nearly all isolates, host-specific genes are induced in only a small fraction of the pathogen population. This supports the hypothesis that for these highly compatible hosts, *B. cinerea* may rely predominantly on general lesion associated mechanisms, making host-specific transcriptional adaptation less critical for successful infection. To investigate whether host-specific genes organize into coregulated transcriptional modules, we constructed coexpression networks. Strongly connected networks were observed in Chard, Lettuce, Bean, Cucumber, and Spinach, whereas Cowpea, Squash, Pepper, and Sunflower had smaller networks (*SI Appendix*, Fig. S23). These 10 networks include a total of 434 genes, of which only 137 had functional annotations. Among the annotated set, several genes were associated with known or putative virulence functions (Dataset S9). Chard networks included a high number of glycoside hydrolase family genes, along with MFS transporters. In Lettuce, beta-lactamase genes were identified, while the Bean network included *BCIN16G05040* (*BcPKS16*), a polyketide synthase involved in secondary metabolism (46). In Spinach, *Bcsdr2*, previously implicated in hyphal growth and pathogenicity on Strawberry and Tobacco, is present (47). NADPH oxidase *BcNOxD* was identified in the Sunflower-specific network (48). These findings suggest that *B. cinerea* expresses potential virulence networks in host-dependent manners.

### Similar Genomic Distribution But Differential Pressure on General vs. Host-Specific Gene Sets.

To start drawing hypotheses for the different genomic properties and evolutionary pressures influencing the genes associated with host-specific, general lesion, or low entropy expression patterns, we assessed their genomic diversity and chromosomal distribution. First, we analyzed whole-genome sequencing data (49) for the isolates and estimated the nucleotide diversity of all genes. This analysis identified that the average nonsynonymous (π_N_) and synonymous (π_S_) substitution ratio in protein-coding genes typically occupies a range from 0.15 to 0.24 with a long right tail (Fig. 4*D*). This suggests that most genes in the pathogen are under purifying selection. Comparing the general lesion, host-specific high entropy, and constitutive low entropy gene sets to a permutation-based confidence interval revealed that host-specific (high entropy) genes are less constrained by purifying selection than low entropy and general lesion-associated genes (Fig. 4*D*). This agrees with their host-specific role as any purifying selection should only occur when pathogen is on the specific host for the gene to be expressed and functional. In contrast, the general lesion genes should be under selection when infecting all hosts.

In a number of fungal pathogens, host-specific genes are often clustered within a genome. To test whether the genes associated with host-specific high entropy, general lesion, or low entropy expression patterns exhibited spatial clustering or genomic compartmentalization, we analyzed the chromosomal distribution of these three gene sets. In contrast to other fungal pathogens that display a “two-speed” genome architecture, where adaptive genes are concentrated in gene-sparse, fast-evolving regions (50–52), all three gene classes in *B. cinerea* are broadly dispersed across the genome, with only minor evidence of localized clustering (*SI Appendix*, Fig. S24). This diffuse distribution supports a model in which regulatory plasticity, rather than structural genome compartmentalization or gene presence/absence, underlies host-responsive transcriptional dynamics in *B. cinerea*.

## Discussion

Generalist pathogens such as *B. cinerea* can infect a wide range of plant species, but the molecular strategies enabling this breadth remain elusive. By leveraging a factorial design that incorporates both extensive pathogen diversity and a phylogenetically broad host panel, we identified a modular strategy underpinning generalism that combines general lesion-associated genes and plastic, host-inducible gene modules that respond to specific host environments. This framework supports a model in which specific host-driven transcriptional plasticity in response to the diverse hosts allows *B. cinerea* to infect a broad host range.

### Host Specificity via Plastic Host-Inducible Genes.

Transcriptomic profiling coupled with entropy-based classification identified a subset of genes with strong host-specific expression patterns. These genes are present in nearly all isolates likely explaining why nearly all *B. cinerea* isolates can infect all tested hosts without the requirement of strict host specialization. This model of host specificity distinguishes *B. cinerea* from pathogens such as *F. oxysporum* and *Verticillium dahliae*, where host range is determined by the presence/absence variation of lineage-specific effectors or accessory chromosomes (53–55).

While these host-specific genes do not show much presence/absence variation, they do differ across the isolates in their plastic response to the host. This agrees with previous work showing that variation of in planta gene expression in *B. cinerea* is due to variation in trans-regulating networks that influence plasticity elements (28, 34). Thus, variation in host specificity appears to derive from genetic polymorphisms in the detection and response to the hosts. evidence points to plant metabolites as potential triggers for these regulatory cascades creating host-specificity. For instance, resveratrol from the grapevine induces *BcLCC2* (56), α-tomatine from tomato upregulates glycosyltransferases and RTA1-like transporters (57), capsidiol from solanaceous plants activates specific detoxification genes (58), and Brassicaceae glucosinolate derivatives upregulate transporter families (59). This leads to a model where the host specificity of a *B. cinerea* could depend on the phylogenetic distribution of the chemicals ranging from largely family specific like glucosinolates to others that are widely but sporadically present, like saponins or cyanogenic glucosides.

Agreeing with this hypothesis, the above-mentioned detoxification genes fall into the “intermediate entropy” category, neither fully conserved nor strictly host-specific. Their variable expression across hosts would provide a flexible layer, tuned by host cues, allowing *B. cinerea* to adapt infection strategies across rapidly evolving hosts without requiring permanent genetic changes. Such graded responsiveness may be key to enabling generalism while preserving host compatibility. Further work is required to fully understand how *B. cinerea* senses and detects particular hosts.

### Core Metabolism Underlies General Lesion Potential.

Alongside host-specific plasticity, we identified 287 genes whose expression was significantly correlated with isolates’ general lesion-forming potential across hosts. These genes formed a coregulated module enriched in peroxisome function, lipid breakdown, and core metabolic functions and another module with solely members of the *Botcinic acid* biosynthetic genes. Unlike host-inducible genes, these virulence genes were expressed across all hosts but varied in magnitude across isolates. Given that these genes form largely coregulated modules, it suggests that their variation is also shaped by variation in the trans-regulatory signaling networks controlling these core modules (28). As core metabolism underpins fundamental processes like growth, stress tolerance, resource acquisition, and resource allocation, even subtle differences in this module could significantly affect disease severity. Elevated expression may also reflect faster growth kinetics rather than direct virulence functions. Disentangling causal roles from correlated metabolic signatures will require targeted functional assays.

### Comparison with Specialist Pathogens and Two Speed Genome Models.

Our findings differ from the two-speed genome architecture described for many hosts specialized pathogens, where rapidly evolving effector genes cluster in repeat-rich compartments and core genes lie in conserved regions (50–52). Current and previous studies suggest that *B. cinerea* lacks such genome compartmentalization as virulence genes are distributed genome-wide and there is no strong clustering for host-specific modules (*SI Appendix*, Fig. S20) (31, 36, 60) This suggests that adaptation to diverse hosts depends on distributed regulatory variation rather than by a strongly compartmentalized genome with rapid virulence gene evolution.

This architecture may reflect the pathogen’s evolutionary history. While specialist interactions often arise from ancient coevolution, phylogenetic dating places the origin of the *Botrytis* lineage at ~10 to 20 Mya (61, 62) long after the radiation of core eudicot hosts (~100 to 120 Mya) (63). This temporal mismatch supports a model of ecological fitting, in which *B. cinerea* expanded onto already divergent host lineages through broad compatibility mechanisms. The ability of the reference isolates *B05.10* to infect basal eudicots further supports this interpretation (23–25). Unlike biotrophs or specialists that often undergo metabolic streamlining and evolve auxotrophies (64), this expansion likely required *B. cinerea* to retain a broad, autonomous metabolic capacity. Consequently, its facultative lifestyle favors polygenic, regulatory variation in broadly shared genes rather than the genomic compartmentalization or metabolic dependency seen in specialists.

### Limitations and Future Direction.

While this study provides a robust transcriptomic framework for plasticity based generalism hypothesis future work needs to prioritize functional validation. A deeper analysis across additional plant families, tissues, and environmental conditions will be needed to measure how broadly this hypothesis functions or if there is a sampling bias. Additionally, *B. cinerea* mutants within the high-entropy, host-specific genes are needed to test if virulence is compromised on only the associated host species. Further, the genes enabling plastic host responses need to be identified and functionally validated. Additionally, while we propose *B. cinerea* as a model for plastic generalism, it is unclear how this equivalent strategy extends to other broad-host-range necrotrophs such as *S. sclerotiorum*, (22). Expanding similar experimental designs to other generalist pathogens will help resolve this question. Finally, extending to a broader sampling across additional plant families, tissues, and environmental conditions will be needed to measure how conserved these plastic infection strategies are across the eudicots and beyond. Together, our results position *B. cinerea* as a transcriptionally plastic generalist that combines a flexible, host-inducible repertoire with isolate-specific variation in the regulation of core metabolism. This modular infection strategy enables broad host compatibility while allowing quantitative tuning of virulence. Future work should focus on functional validation of both gene modules and dissection of the regulatory polymorphisms, particularly trans-acting factors, that underlie this plasticity. Expanding this framework to additional hosts, tissues, and environmental conditions, alongside deeper exploration of host-derived cues and host-side dynamics, will enhance our understanding of generalism and its ecological maintenance.

## Materials and Methods

All experimental procedures and analytical methods are described in detail in *SI Appendix*, *Materials and Methods*. This includes plant growth conditions, selection of host genotypes and *B. cinerea* isolates, detached leaf infection assays, lesion quantification, statistical modeling of lesion traits, RNA-seq library preparation, fungal transcriptomic profiling, entropy-based gene classification, coexpression network construction, comparative in planta vs. in vitro expression analysis, and evolutionary inference of selection using πN/πS estimates from population genomic data.

## Supplementary Material

Appendix 01 (PDF)

Dataset S01 (XLSX)

Dataset S02 (XLSX)

Dataset S03 (XLSX)

Dataset S04 (XLSX)

Dataset S05 (XLSX)

Dataset S06 (XLSX)

Dataset S07 (DOCX)

Dataset S08 (XLSX)

Dataset S09 (XLSX)

Dataset S10 (XLSX)

Dataset S11 (XLSX)

Dataset S12 (XLSX)

Dataset S13 (XLSX)

Dataset S14 (XLSX)

## Data Availability

The RNA-seq data have been deposited in the NCBI Sequence Read Archive (SRA) under BioProject ID PRJNA1217477 (65). Raw sequencing data from the pilot experiments used to optimize the sampling time point are available under BioProject PRJNA1428298 (66). All data supporting the findings of this study are included in the main article and/or the supporting information.
