# Supplementary material for: Combined generalist and host-specific transcriptional strategies enable host generalism in the fungal pathogen Botrytis cinerea
Source: Proc Natl Acad Sci U S A. 2026 May 19;123(21):e2521414123. doi: 10.1073/pnas.2521414123 (PMC13214029; doi:10.1073/pnas.2521414123)
Supplement: Supplementary file 1 — Appendix 01 (PDF) [file pnas.2521414123.sapp.pdf]

Supporting Information for

Combined generalist and host-specific transcriptional strategies enable host generalism in the fungal pathogen *Botrytis cinerea*

Authors: Ritu Singh[1], Anna Jo Muhich[1], Cloe Tom[1], Jack McMillan[1], Karishma Srinivas[1], Lucca Faieta[1], Celine Caseys[1], Daniel J Kliebenstein[1, *]

[1] Department of Plant Science, University of California, Davis, CA, USA

***Corresponding author:**

Daniel J. Kliebenstein

Professor, University of California, Davis, CA, USA

kliebenstein@ucdavis.edu

This PDF file includes:

- **SI Materials and Methods**
- **Supplementary Figures S1 to S24 with their legends**
- **SI Dataset legends**
- **SI References**

SI Appendix Materials and Methods

Plant material and growth conditions

To test how genetic diversity within *B. cinerea* as well as across and within host plant species shapes the disease outcome, host species from a total of eight eudicot orders were included: three Asterids - Apiales, Asterales, and Solanales; one Super-Asterids - Caryophyllales; and four Rosids - Cucurbitales, Fabales, Brassicales, and Malvales (**Figure 1**). Within each order, the species sampled belonged to the same family to determine if virulence strategies are conserved among closely related hosts or if significant variation exists even between phylogenetically similar species. Two representative plant species were selected per order, except for the Malvales, which was represented by a single species, *Alcea rosea* (Hollyhock). The species used were: *Petroselinum crispum* (Parsley) and *Apium graveolens* (Celery) from Apiales; *Helianthus annuus* (Sunflower) and *Lactuca sativa* (Lettuce) from Asterales; *Capsicum annuum* (Pepper) and *Solanum lycopersicum* (Tomato) from Solanales; *Spinacia oleracea* (Spinach) and *Beta vulgaris* subsp. *vulgaris* (Chard) from Caryophyllales; *Brassica rapa* (Mustard) and model plant *Arabidopsis thaliana* from Brassicales; *Vigna unguiculata* (Cowpea) and *Phaseolus vulgaris* (Common Bean/Bean) from Fabales; and *Cucumis sativus* (Cucumber) and *Cucurbita pepo* (Squash) from Cucurbitales. These hosts were selected to sample from annual diploid species for which a reference genome is available, and for which there were reports of field infections by *B. cinerea*.

For the leaf infection assays, four genotypes for each plant species were selected to sample intraspecific genetic diversity (**Dataset S3**). Genotypes were obtained from the USDA-Germplasm Resources Information Network (GRIN), the UC Davis Tomato Genetics Resource Center, the Center for Genetic Resources (CGN) in the Netherlands, and the Michelmore lab from UC Davis. The genotype selection was made in consultation with experts of each crop to maximize genetic diversity based on available information. For clarity, all plant species are referred to as “species” or “hosts” throughout the manuscript.

For the co-transcriptomic study, a subset of five eudicot orders was selected: Asterales and Solanales (Asterids), Caryophyllales (Super-Asterids), and Cucurbitales and Fabales (Rosids), using the same two species per order as described above **(SI Appendix, Figure S1)**. This subset was chosen because these species generated reliable fungal reads at 48 hpi, were diploid and easy to grow under controlled conditions, and supported consistent *B. cinerea* infection necessary for large-scale co-transcriptome analysis. To ensure experimental feasibility and enable cross-species comparisons within the broader framework, only one representative genotype per species was used **(Dataset S3)**. This genotype was chosen based on the lesion data, representing an intermediate lesion development for that host species (**Dataset S1-S2**).

For both the lesion phenotyping and transcriptomic analysis, all plants were grown in controlled environment chambers in pots containing Sunshine Mix #1 (Sun Gro Horticulture, Agawam, MA, USA). Growth conditions were standardized at 20°C with a 16-hour photoperiod and light intensity

of 100–120 µmol m² s¹. Plants were watered every two days with deionized water for the first two weeks, followed by a nutrient solution containing 0.5% N–P–K fertilizer in a 2–1–2 ratio (Grow More 4–18–38).

B. cinerea isolate collection and culture

For all assays, we used 72 *B. cinerea* isolates collected from 14 different host plants and a range of geographical locations (**Dataset S11**). Our isolate collection contains no significant genetic stratification by either geographic or host species origin (1, 2). All these isolates were previously characterized across eight eudicots (2) and on Arabidopsis (3, 4). The 72 *B. cinerea* isolates sample the range of genetic diversity, virulence, and host specificity observed within a larger population of 96 isolates (2, 5).

All the isolates are maintained as conidial suspensions in 60% glycerol at -80 °C for long-term storage. For experiments, spores were grown on potato dextrose agar (PDA) plates by diluting 1:10 (v/v) of glycerol stocks in grape juice and incubated at 21 °C for two weeks.

B. cinerea lesion assays

To enable *B. cinerea* lesion assays comparability across diverse plant species, the standard detached leaf assay was used, using adult leaves as a common organ (2, 6, 7). Detached leaf infections have been shown to correlate well with whole-plant infection assays in various pathosystems (7–9). This approach enabled testing of a large collection of *B. cinerea* isolates while maintaining uniform assay conditions across diverse plant taxa. To ensure consistency in the developmental stage across species, fully expanded mature leaves were sampled during the vegetative phase (4-8 weeks after planting, depending on species-specific growth rates) before the onset of bolting/flowering. This approach minimized ontogenetic variation and ensured comparable leaf maturity across all species used in the experiments. Given the scale of the experiment, it was not possible to introduce developmental or ontogenic variation into the analysis.

Detached leaf assays were performed as previously established (2, 7, 10). In brief, spores were extracted in sterile water, counted using a hemacytometer, and diluted in 50% grape juice to a final concentration of 10 spores/µL. Grape juice, a complex mixture of sugars and micronutrients, was used as the inoculum medium to promote consistent germination across isolates. This alleviates issues where *B. cinerea* has genetic variation impacting germination on single sugar sources (7, 11–13). For infection, leaves were placed on trays containing 1 cm of 1% Phytoagar™ (Plant Media), which maintained leaf hydration and physiological activity during the experiment. Leaves were inoculated with 4 µL droplets (40 spores). Grape juice was used as a control. To prevent premature germination during inoculation, spore suspensions were kept on ice and gently agitated to maintain a uniform distribution of spores. Inoculated trays were placed under humidity domes and incubated under constant light. Each isolate × plant genotype combination was replicated three times per experiment in a randomized complete block design. The entire experiment was independently repeated twice, yielding six biological replicates per host-isolate interaction. The

lesion area, a quantitative measurement of the host-pathogen interaction, was assessed at 72- and 96-hours post-inoculation (hpi).

For transcriptome analysis, the same setup was used with a single genotype per plant species (**Dataset S3**). Infection of each isolate on each plant was done as described above (3, 4). Leaf disks surrounding the infection site were harvested at 48 hpi using a cork borer of 1.20 cm diameter, ensuring uniform tissue collection across species. At this timepoint, the lesions were not extensively developed, and the majority of the samples were living plant cells. Samples were flash-frozen for RNA-seq library preparation. To confirm infection success, leaves from the same plants, spore batches, and trays were kept at room temperature until 72 hpi to monitor and ensure proper lesion development.

Lesion measurement and data quality control

Lesion area, a quantitative measure of host-pathogen interaction, was assessed at 72 and 96 hpi. For species that developed large lesions rapidly (Tomato, Sunflower, Lettuce, Arabidopsis, and Mustard), measurements were taken only at 72 hpi to avoid any constraints on lesion development caused by tissue availability. Infection trays were photographed using a Canon T3i camera (18 MP) at a fixed distance and consistent top lighting, which ensured a consistent image quality. Images were analyzed with an R pipeline (14), which converted images to hue/saturation/value (HSV) color space and applied species-specific thresholds to detect lesions. Leaf and lesion masks were generated, manually curated, and the lesion areas were quantified in pixels. A scale included in each image allowed conversion to square millimeters. The dataset comprised 24,966 lesion measurements at 72 hpi and 17,520 at 96 hpi (**Dataset S1-S2**). Data were filtered to remove technical failures (e.g., droplets without *B. cinerea* growth) as per (2).

Lesion modeling

The effect of host and pathogen genetic variation on lesion area was analyzed using a two-stage analysis approach, which is a recommended approach for large multi environment designs where full single stage models become computationally prohibitive (15–17). This strategy provides stable estimates of variance components across hierarchical biological levels while correcting experimental variation before meta-analysis.

In the first stage, we modeled the log-transformed lesion area for each host species individually according to the host (genotype), pathogen (isolate), and their interactions while accounting for experimental design using linear mixed models implemented in the lme4 package (18).

Linear mixed model: Log-transformed Lesion Area ~ Genotype + Isolate + Genotype*Isolate + (1|Experiment) + (1|Tray) + (1|Plant)

Here, experiment (experimental replicate), tray (microenvironment containing subsets of leaves within a randomized complete block design), and plant (individual plant identity from which detached leaves were collected) were treated as random effects. Plant genotypes and *B. cinerea*

isolates were treated as fixed effects (6, 14). Diagnostic residual-fitted plots for these models are provided in **SI Appendix, Figure S5**. Given that at the sampling time points, the lesions had not consumed most of the leaf, the leaf area does not influence lesion area development (2). Corrected log transformed least square means (LS-means) for lesion area for each host genotype x *B. cinerea* isolate combination were extracted from the models (19). Log transformation was applied prior to modeling to correct mean-dependent variance heterogeneity, as supported by residual diagnostics. These LS-means represent the adjusted phenotypic values after correcting for experimental environmental variation (Tray, Plant, Experiment) and were used as the response variable for downstream analyses.

In the second stage, we constructed a multi-host meta-model to evaluate the relative contributions of host phylogenetic diversity and pathogen virulence to lesion development across host species. We utilized the log-transformed LS-means generated in Stage 1 in a nested linear model framework. Host plants were hierarchically nested by taxonomic levels (Clade > Order > Species > Genotype), and *B. cinerea* isolate was treated as a fixed effect. Interaction terms were included to assess isolate-by-host lineage effects on lesion development. Although the model incorporated plant phylogenetic structure via taxonomic hierarchy, it did not explicitly account for divergence time.

The multi-host model was specified as: Log-transformed Mean Lesion Area (LS-means) ~ Isolate + Clade/Order/Species/Genotype + Isolate*(Clade/Order/Species).

Because there is a single mean per Isolate by Genotype combination, the residual term captures both variance associated with the Isolate by Genotype interaction and any experimental stochasticity.

Model sensitivity was assessed by repeating the entire workflow, from single species models to the multi host meta model, using non transformed lesion area values (**SI Appendix, Figure S3A, S4, 6A-C**). While log transformed data improved residual diagnostics with moderate shifts in absolute variance estimates, the rank order pattern and statistical significance remained unchanged in both single species models as well as multi host model (**SI Appendix, Figure S3A-B, S6C, and 1A**). To enable a fuller inspection of the data and models, log transformed results are presented in the main figures and non-normalized data analyses are provided in the supplementary material. This enables the reader to conduct a direct comparison and independent evaluation of how the models are or are not affected by normalization.

To further evaluate the robustness of the above mentioned two-stage log transformed and non-transformed framework, we implemented an additional random effect subtraction analysis. We fitted the full mixed model on raw lesion measurements, extracted random intercepts for Experiment, Tray, and Plant, and subtracted these values from the observed lesion area to remove experimental design effects. To ensure this model was computationally feasible and comparable to the two-stage framework given the extreme size of the data matrix, we omitted the Genotype x

Isolate interaction term. The adjusted response values were then analyzed using the corresponding fixed effect model containing Isolate, nested phylogenetic structure, and their interaction. This subtraction-based approach produced the same hierarchical order of variance components across the hosts as the two-stage framework, confirming that host phylogenetic structure dominates lesion variation independent of experimental random effects (**Figure 1A, SI Appendix, Figure S6C-D**). We noted that while Isolate effects remained statistically significant in this model, their relative contribution was reduced in comparison to two-stage model. We attribute this to our experimental design structure: because Isolate effects are estimated at the individual plant level in Stage 1 but aggregated in Stage 2, the subtraction method likely redistributes some Isolate-associated variance into the residual.

We also attempted to fit a full single stage model incorporating all raw lesion measurements across hosts, isolates, genotypes, and experimental factors simultaneously. However, due to the size and complexity of the dataset, these models were computationally infeasible and failed to converge. The two-stage and subtraction based approach therefore represents a practical and statistically robust solution for this dataset. Consistent with published evaluations of linear mixed models, the high degree of replication in our dataset stabilizes parameter estimates even under moderate departures from normality (20).

Hierarchical clustering was performed on standardized LS-means of lesion area (Z-score transformed; centered and scaled for each isolate) utilizing Euclidean distance as the similarity metric and the complete linkage agglomeration method. Cluster stability was estimated via multiscale bootstrap resampling (n = 1,000) using the *pvclust* package (2, 21, 22); clusters with an Approximately Unbiased (AU) p-value $\geq$ 95% were considered strongly supported. Heatmaps were visualized using the pheatmap package (21). To minimize bias from missing data, six isolates that failed to sporulate or grow in time for the detached leaf assay of certain species were excluded from this analysis.

General lesion vs host-dependent lesion

Previous work has shown that it is possible to use lesion area across diverse hosts to estimate the general lesion formation capacity of a *B. cinerea* isolate in addition to measuring the isolate's lesion formation potential on individual hosts (host lesion) (23). To estimate the general and host lesion contributions for the *B. cinerea* isolates, we used linear mixed models. General lesion was estimated by modeling the log transformed LS-means of lesion area across all species using the model: Log Mean Lesion Area ~ Isolate + (1 | Species) (23). To avoid overfitting and ensure that the general lesion estimate was not biased by performance on the focal species, we implemented a leave-one-species-out approach, whereby the species being evaluated was excluded from the model during its general lesion estimation. This step was critical because including the focal species could inflate the general lesion estimate on that host, especially when regressing host-dependent lesion values against the general lesion, due to circularity. Here, isolate was treated as a fixed effect to estimate isolate-specific lesions across hosts, while fifteen species were treated as

a random effect to account for interspecies variability. The average lesion across all hosts for each isolate was obtained as an estimate of general lesion potential.

To estimate each isolate's lesion-forming potential on individual host species (host-dependent lesion), we fit a separate model for each species: Log Mean Lesion Area ~ Isolate + (1 | Genotype). Within each species, the four genotypes were modeled as a random effect to capture within-species genetic variation, while *Botrytis* isolates remained a fixed effect. This model estimated isolate performance on a specific host, adjusting for genotype-level variation.

To quantify deviations in isolate performance from their expected general lesion value, we modeled the relationship between host dependent lesion and general lesion for each isolate using the linear regression: host-dependent lesion ~ general lesion. For each isolate, we calculated the residual standard error, sigma, from this regression as: $\sigma = \text{sqrt}(\Sigma \text{ residual}^2 / (n - 2))$ where n represents the number of host species sampled for that isolate. Sigma reflects the square root of the mean squared residual adjusted for degrees of freedom and provides a single descriptor of host range variance. Higher sigma values indicate greater deviation from the general lesion trend across hosts, consistent with increased host specific variability in lesion formation.

RNA-Seq library preparation, sequencing, and mapping

B. cinerea infected leaf tissues from ten plant species, along with grape juice control, were sampled at 48 hpi for transcriptome analysis as described above. This timepoint chosen for sampling was optimized based on two preliminary pilot experiments. An initial multi-species survey at 16 hpi revealed insufficient fungal transcript recovery for most hosts. Subsequently, a time-course pilot conducted at 24, 30, 36, and 48 hpi demonstrated that the 48 hpi window yielded the highest proportion of fungal-mapped reads across all species while maintaining tissue integrity. Raw data for these pilot scoping runs are available for independent query via the NCBI SRA (BioProject PRJNA1428298).

For the main study, a total of 2190 mRNA libraries were prepared for paired-end sequencing using the Element Biosciences AVITI PE150 platform (DNA Technologies Core, Davis, CA). RNA-Seq libraries were prepared according to the previous method (24) with minor modifications (3). Briefly, infected leaves were immediately frozen in liquid nitrogen and stored at -80°C until processing. RNA extraction was conducted by re-freezing samples in liquid nitrogen and homogenizing by rapid agitation in a bead beater, followed by direct mRNA isolation using the Dynabeads mRNA Direct purification kit (Invitrogen). First and second-strand cDNA were produced from the mRNA using an Invitrogen Superscript III kit. The resulting cDNA was fragmented, end-repaired, A-tailed, and barcoded as previously described (24). Libraries were size-selected for ~300 bp and pooled in 96-sample batches for sequencing at the UC Davis Genome Center (DNA Technologies Core, Davis, CA).

Fastq files from individual sequencing lanes were separated by adapter index into individual RNA-seq library samples. Raw RNA-seq reads from individual libraries were subjected to quality

control using MultiQC v1.15 to assess overall read quality metrics, including per-base sequence quality and the presence of overrepresented sequences (25). Adapter index and low-quality bases were trimmed using Trimmomatic v 0.39 (26). Cleaned reads were first mapped to the host reference genome (**Dataset S12**) using Hisat2 version 2.2.1 with phred33 quality scores and modified alignment parameters to account for mismatches at the read ends (27). The remaining unmapped reads were subsequently aligned to the *B. cinerea* B05.10 isolate reference genome using the same Hisat2 version and parameters (28). Gene counts were pulled from the resulting SAM files using SAMtools (29) and converted to BAM files. Custom R scripts were used to summarize counts across gene models to reduce the overrepresentation of genes with multiple splice variants.

B. cinerea gene expression analysis

Raw read counts for *B. cinerea* genes were processed and normalized using R (v4.3.1) and the edgeR package (v3.42.4) (30). To remove lowly expressed genes while preserving biologically relevant, isolate- or host-specific transcripts, we applied a minimal expression filter, retaining genes with a count-per-million (CPM) value of > 1 in at least two samples per host using the cpm function in edgeR. In this case, CPM refers solely to the reads that map to *B. cinerea* in the sample. This threshold minimizes background noise without excluding lineage-specific or conditionally expressed genes. Gene counts passing this filter were normalized using the Trimmed Mean of M-values (TMM) method. Library size-corrected CPM values were extracted for downstream analyses.

To estimate *B. cinerea* transcript abundance in infected tissue, we calculated the percentage of total reads (host plus pathogen) that mapped to *B. cinerea* genes (% *B. cinerea* reads) for each RNA-Seq sample. This was done by dividing the number of reads mapped to *B. cinerea* genes by the total mapped reads per sample and multiplying by 100:

% *B. cinerea* reads = (*B. cinerea* mapped reads / Total mapped reads) × 100.

This value was computed independently for each plant species and tested as a proxy for fungal biomass.

To test how the *B. cinerea* transcripts vary across the diverse host plants, the isolate variation, and their interaction, we fitted a linear mixed model for each transcript.

Linear mixed model: CPM ~ (1 | *B. cinerea* isolate) + (1 | Host) + (1 | Isolate:Host)

where CPM denotes the normalized counts per million for each transcript, isolate represents 72 *B. cinerea* isolates, host represents the 10 eudicot species, and the interaction term accounts for host-pathogen interactions. Models were run separately for the full dataset as well as subsets at the clade and order levels to explore broader phylogenetic patterns. Modeling was not performed at the individual species level due to the presence of only one genotype per species.

For each transcript, broad-sense heritability (H^2) was calculated as the proportion of total variance explained by genetic sources (isolate, host, and their interaction) (31). The resulting H^2 values were visualized using ternary density plots generated with the ggtern package (32) in R (v4.3.1), which illustrate the relative contribution of each source to transcriptomic variation.

Negative binomial modeling of transcript variation

To quantify transcript-level effects of host, isolate, and their interaction while accounting for overdispersion in RNA-seq data, we fitted gene-wise negative binomial generalized linear mixed models using glmmTMB package in R. Initial implementation of gene-wise negative binomial (log link) generalized linear mixed models (nbGLM) failed to converge for a substantial fraction of genes due to the prevalence of transcripts expressed only in a limited subset of hosts or isolates. These true biological zeros led to instability in dispersion estimation and model optimization. To stabilize model fitting while retaining zero-expression genes, a small constant of 0.5 was added to CPM values prior to analysis. This offset mitigates computational issues associated with zero counts while preserving relative expression structure across samples. After applying this adjustment, only 98 genes failed to converge.

For each gene, the following model was fitted:

CPM ~ species + isolate + species*isolate + (1 | tray) + (1 | sequencing_batch)

where species represent host species, isolate represents *B. cinerea* isolate, and tray and sequencing batch were modeled as random effects to account for experimental structure. A negative binomial distribution with a log link was used to account for count dispersion.

Type II Wald chi-square tests were used to evaluate fixed effects. For each gene, we extracted sums of squares, degrees of freedom, chi-square statistics, and p-values. Variance components for fixed effects were derived from the conditional variance-covariance matrix and expressed as the proportion of total modeled variance. Estimated marginal means for isolate-by-species combinations were calculated using the emmeans package (33, 34) and are provided in **Dataset S14**. Full statistical outputs for all genes, including Wald chi square statistics, degrees of freedom, *p* values, and variance partitions, are provided in **Dataset S13.**

Shannon Entropy calculation and classification of genes

Shannon entropy is commonly used to distinguish constitutively expressed genes from condition- or tissue-specific genes (35–37). In the context of plant-pathogen interactions, we applied Shannon entropy to quantify variability in *B. cinerea* gene expression across host species using the BioQC package (35). Genes with low entropy show similar expression across the hosts and were classified as conserved. In contrast, high entropy genes are highly variable across hosts and were used to classify host-specific genes, where gene expression was limited to one or a few specific hosts.

Entropy was calculated directly from linear-scale, library-size-normalized CPM proportions (35). We strictly avoided log-transformation or model-derived estimates for these calculations, as Shannon entropy is defined by the probability distribution of relative abundances. Following published best practices (35) preserving the linear proportional relationships is essential; non-linear transformations can compress the data variance, which flattens the distribution and biases estimates of expression breadth.

To focus on the expression patterns observed across hosts and reduce noise due to the genetic diversity across the 72 *B. cinerea* isolates, we calculated the mean of CPM-normalized expression values within each host species. To validate this method, entropy was also calculated for all 72 isolates, and the resulting gene ranks were concordant across methods. The genes were categorized into low, intermediate, and high entropy using Jenks' natural breaks optimization function (getJenksBreaks in BAMMtools) (38). Jenks natural breaks is a data classification method that partitions continuous values into groups that minimize variation within each group and maximize differences between groups. Based on this clustering, 589 genes had high entropy ($H > 3.25$), 10,828 genes were intermediate ($3.25 > H < 0.56$), and one gene had low entropy ($H < 0.0030$).

To account for the range and distribution of entropy values and allow identification of the host(s) associated with high-entropy genes, gene expression values were log-transformed [$\log_2(\text{CPM} + 1)$] and z-score normalized across host species for each gene individually. A gene was considered highly expressed in a particular host if its z-score exceeded +1, indicating expression at least one standard deviation (SD) above its mean.

Genes with z-scores > +1 SD in only one host were classified as single-host-specific (n = 434), while those with z-scores > +1 SD in two to three hosts were labeled as multi-host-specific (n = 82). Seventy-three genes initially assigned to the high entropy group by Jenks clustering but exhibiting low inter-host variance were excluded based on the z-score > +1 criterion. Each single-host-specific gene was assigned to the host with the highest z-score.

General lesion-associated genes

To identify *B. cinerea* genes associated with the general lesion estimate across diverse host species, we fitted the linear model for each gene:

General lesion ~ Gene expression + Host + Gene expression x Host.

This model tests the relationship between fungal gene expression (CPM value) and general lesion, while accounting for host identity and gene-by-host interaction effects. *P*-values associated with each model term were corrected for multiple testing using the Benjamini-Hochberg false discovery rate (FDR). Genes with FDR-adjusted *p*-values below 0.05 for the main gene expression term in the model (i.e., independent of host or interaction effects) were considered as general lesion-associated genes and used for further analysis.

Co-Expression network analysis of high-entropy, low-entropy, and general lesion-associated genes

Gene co-expression networks were constructed using Pearson correlation coefficients to evaluate potential coordination among different gene classes: host-specific high-entropy genes, conserved low-entropy genes, and general lesion-associated genes.

For the high-entropy networks, only genes with elevated expression (z-score > +1) in a particular host were included in that host's network. For example, a gene showing the highest expression (z-score > +1) in Chard was included exclusively in the Chard-specific network, and its expression across the 72 *B. cinerea* isolates infecting Chard was used to compute pairwise correlations. This approach ensured that each host-specific network captured gene co-regulation patterns uniquely associated with transcriptional responses induced by that particular host.

In contrast, the low-entropy and general lesion-associated gene networks were constructed using expression data across all 720 isolate–host combinations. Given that only a single low-entropy gene was detected by Jenks' clustering, we included 500 genes with the lowest entropy values to low-entropy network. The general lesion-associated network included 287 genes.

For all networks, Pearson correlation coefficients were computed for all gene pairs. The absolute value of the correlation was used as the similarity measure based on the assumption that opposing variation patterns across samples share common regulatory pathways, although we acknowledge that this assumption may not hold in all cases (e.g., due to timing differences in mRNA degradation). Edges were retained if the absolute correlation was ≥ 0.7, a threshold chosen to reflect strong co-expression while minimizing noise (39). Networks were built using the igraph package in R and exported as edge lists and node degree tables for visualization in Cytoscape v3.10. This resulted in a total of 10 host-specific co-expression networks, in addition to the low-entropy and a general lesion-associated gene networks.

Comparison of *in planta* and *in vitro B. cinerea* transcriptomes

To estimate *in vitro* gene expression and compared to *in planta* estimates, we analyzed publicly available RNA-seq datasets of the reference isolate *B05.10* grown in potato dextrose broth (PDB), a media with the same sugar composition as the PDA used in our infection assays. Only control or mock samples were used from three projects: PRJNA1056687 (6–48 h; (40)), PRJNA955032 (0 h; (41)), and PRJNA1173356 (0–24 h), collectively capturing gene expression over multiple growth timepoints. The reads were aligned and normalized with the same pipeline and parameters as mentioned above for *in planta* analysis.

To compare *in vitro* and *in planta* expression, we used only *B05.10* isolate data across ten host species (*in planta*). Comparisons were performed separately for three gene sets: (1) 500 conserved low-entropy genes, (2) all 434 single-host-specific high-entropy genes, and (3) 287 general lesion-associated genes. For each gene within each set, average normalized expression ($\log_2$ [CPM + 1])

was calculated separately across all *in vitro* and *in planta* samples. The absolute difference between average *in planta* and *in vitro* expression values was used to classify each gene into one of three expression categories: (1) Similar expression in both *in vitro* and *in planta* (absolute difference ≤ $\log_2$ unit); (2) Higher expression *in planta* (absolute difference >1 $\log_2$ unit *in planta*); and (3) Higher *in vitro* (absolute difference >1 $\log_2$ unit *in vitro*).

Inference of the strength of purifying selection

To estimate the selective pressure on *B. cinerea* genes, we analyzed the whole-genome sequencing data (PRJNA525902) available for the isolates (42). In short, the Illumina reads were the B05.10 reference genome assembly ASM83284v1 (43) with bwa mem (44). Single nucleotide polymorphisms (SNPs) were extracted with Freebayes (45). The rate of non-synonymous (π_N) and synonymous (π_S) substitutions in protein-coding genes were estimated in SNPGenie (46). To provide a confidence interval of π_N/π_S values to compare the three gene sets, we also analyzed 100 permutations of 500 random genes.

Supplementary Figures

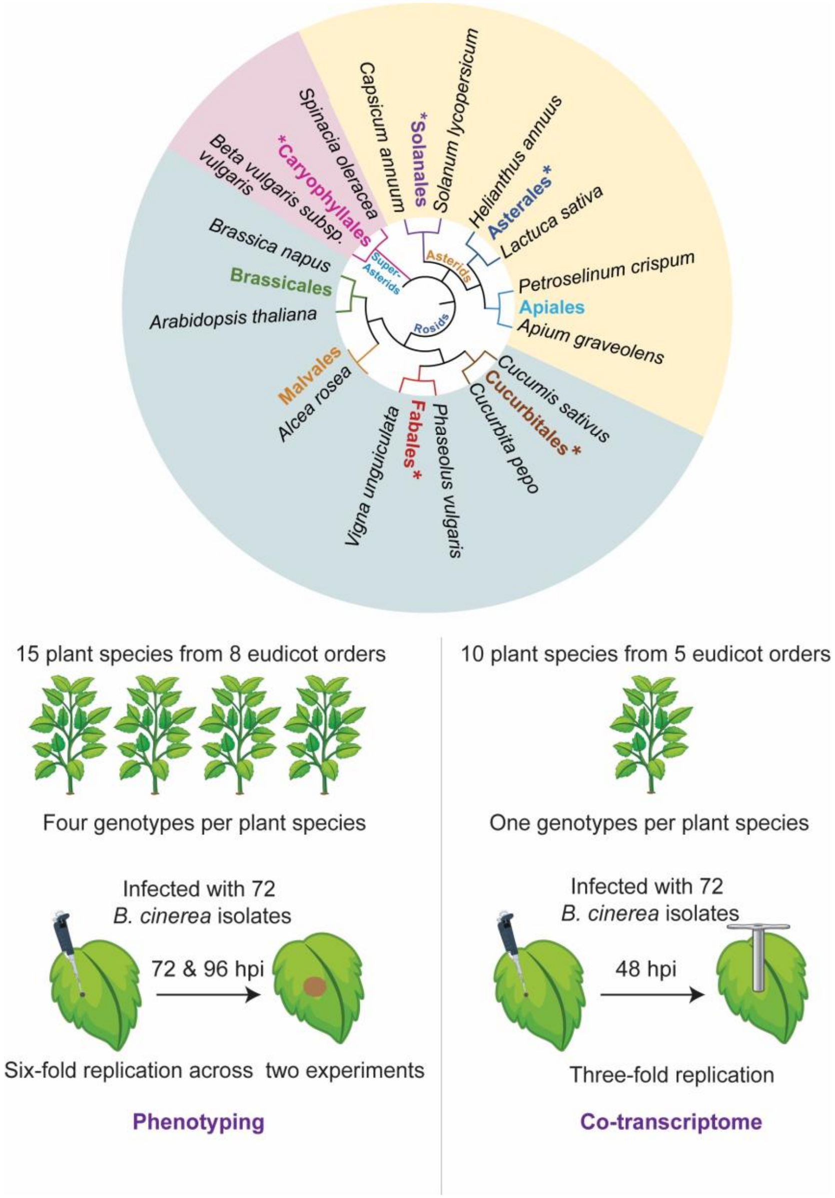

Figure S1: Experimental design for lesion phenotyping and co-transcriptome analysis. We tested the virulence of 72 *B. cinerea* isolates across 15 eudicot plant species from 8 eudicot orders, selected to represent a balanced phylogenetic distribution. Each plant species included four different genotypes used for phenotyping assays. For lesion phenotyping, leaves were inoculated and lesion sizes measured at 72- and 96-hours post-inoculation (hpi) with six-fold replication across two independent experiments. A subset of 10 plant species from 5 eudicot orders (marked with * in the phylogenetic tree) were selected for co-transcriptomic profiling. These species were inoculated with the same 72 isolates, and leaf tissue was harvested at 48 hpi for co-transcriptome analysis, with three-fold replication. Leaf disks were collected using a cork borer of the same size to ensure consistency in tissue quantity across samples.

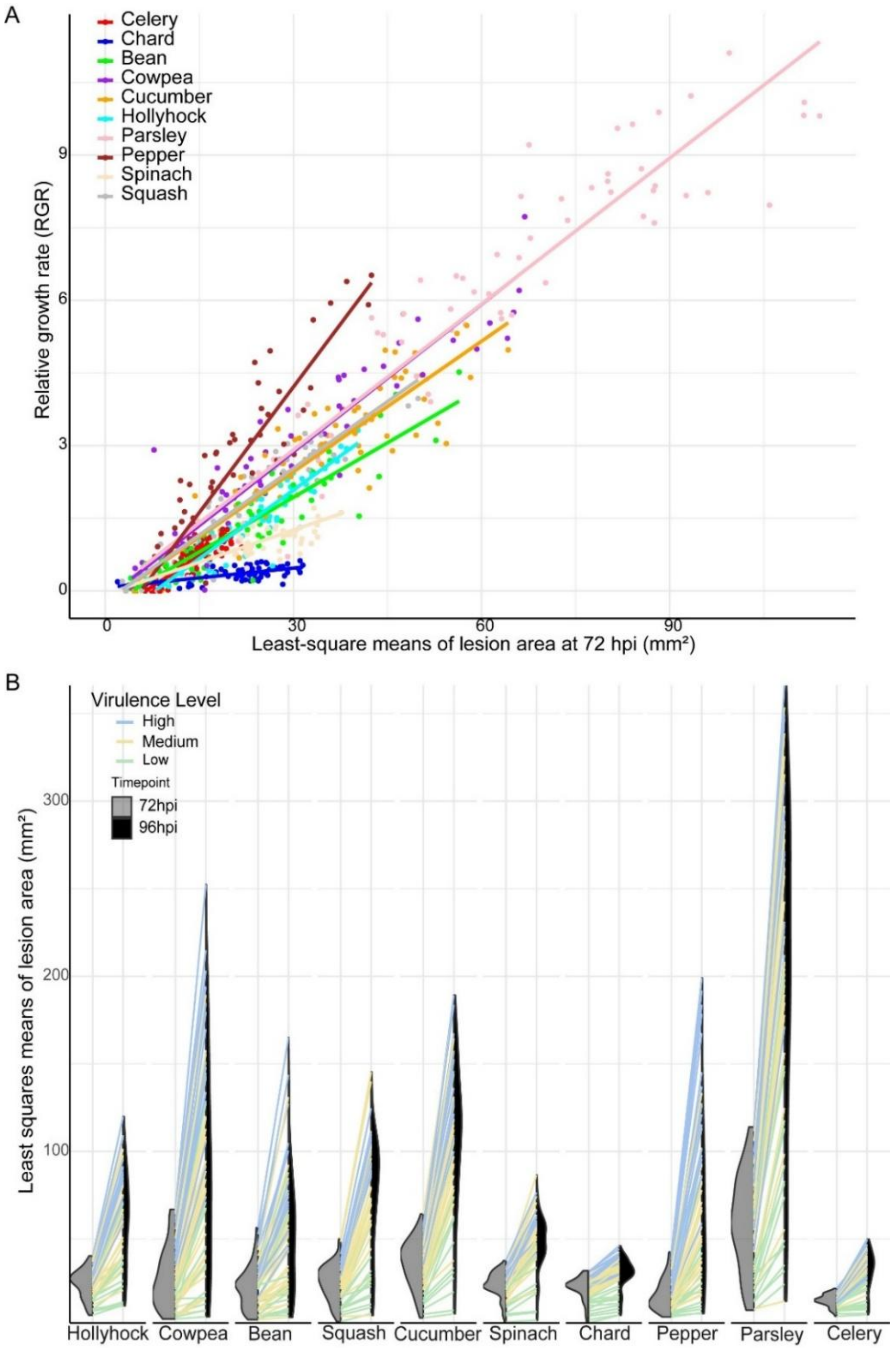

Figure S2. Lesion progression dynamics of *B. cinerea* from 72 to 96 hours post-inoculation (hpi) across 10 eudicot host species. (A) Scatterplot showing the linear relationship between mean lesion area at 72 hpi (LS-mean) and the relative growth rate (RGR) of lesions from 72 to 96 hpi across 10 host species. Each point represents a *B. cinerea* isolate, colored by host species. Solid lines indicate linear regressions fitted separately for each host. The subset of 10 species used for co-transcriptomes was selected for this time-course analysis. **(B)** Half violin plots showing lesion progression from 72 (gray) to 96 (black) hpi across 10 eudicot host species. Lines connect data

points for each *Botrytis cinerea* isolate, which are classified into three virulence categories based on their mean lesion area across all hosts at 72 hpi: high (blue), medium (yellow), and low (green) virulence. Only 10 host species are shown for this time-course analysis due to constraints on lesion development caused by tissue availability.

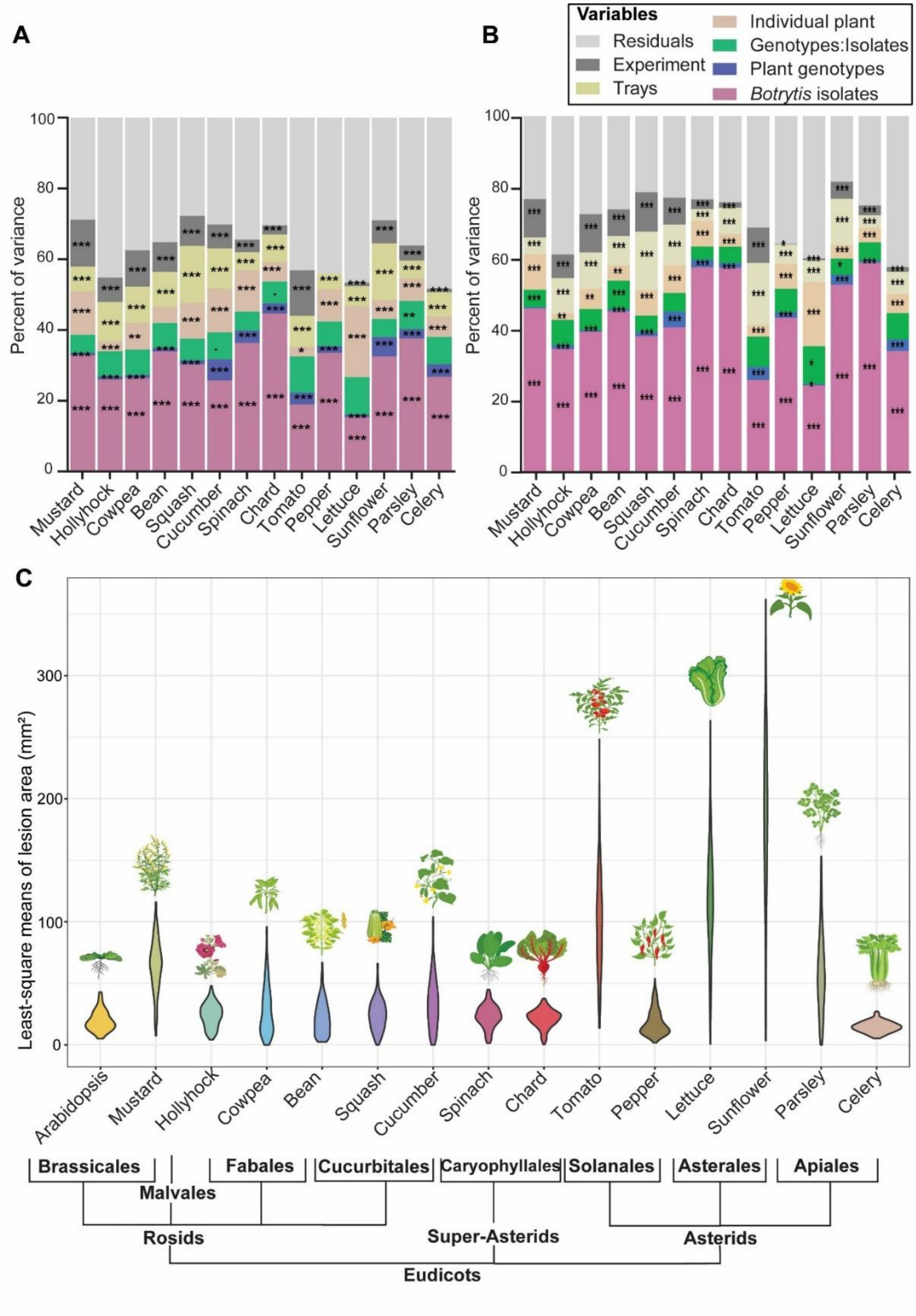

Figure S3. Quantitative variation in host susceptibility to *B. cinerea* across 15 eudicot species at 72 hours post inoculation (hpi). Variance partitioning based on **(A)** raw lesion area. **(B)** log transformed lesion area**.** For panels A and B, the partitioning of experimental and genotypic

variance within individual plant species using linear models of lesion area at 72 hpi is shown. Stacked bar plots show the proportion of total variance in lesion size explained by various experimental factors. Fixed effects include *B. cinerea* isolate (purple), plant genotype (blue), and their interaction (green). Random effects include experimental design factors; individual plant, tray, and independent experiment (beige and gray tones). The model residual is shown in gray. Asterisks indicate the statistical significance of each variance component (*$p < 0.05$, **$p < 0.01$, ***$p < 0.001$). Comparable patterns are observed for raw and log transformed data, indicating robustness of the results. **(C)** Violin plots represent the distribution of average lesion area (mm^2) across 15 eudicot plants infected with 72 *B. cinerea* isolates. A non-scaled phylogenetic tree illustrates the evolutionary relationships among species. Illustrations of representative plant species are shown above each violin plot.

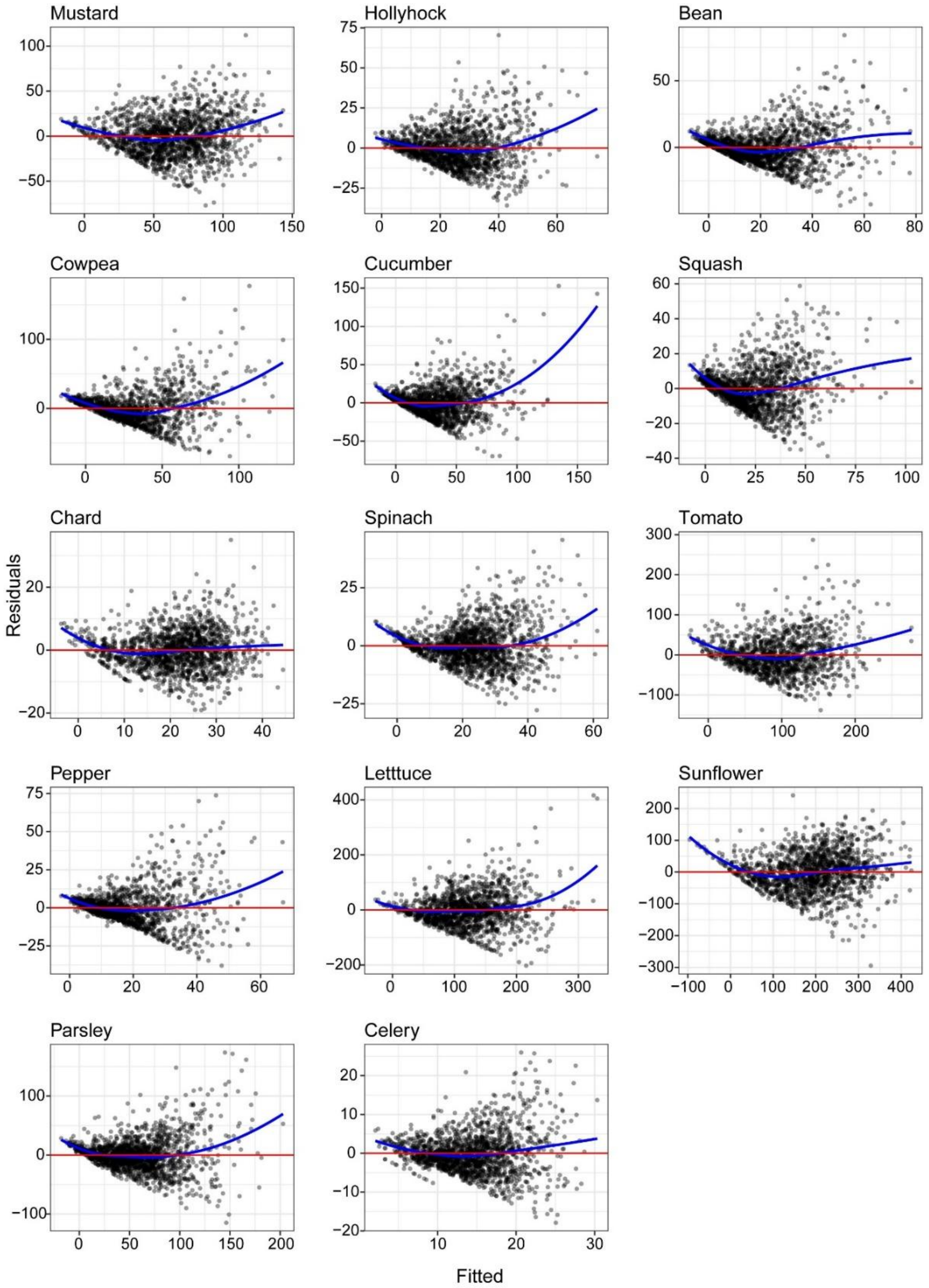

Figure S4. Residuals versus fitted plots from the single species linear mixed models fit to raw lesion data. Scatter plots display the relationship between the fitted values (predicted lesion area in mm²) and the residuals for each of the host species analyzed. Dots represent individual residuals for each biological replicate, while the blue solid line represents a LOESS smoothing curve illustrating the trend of residual means.

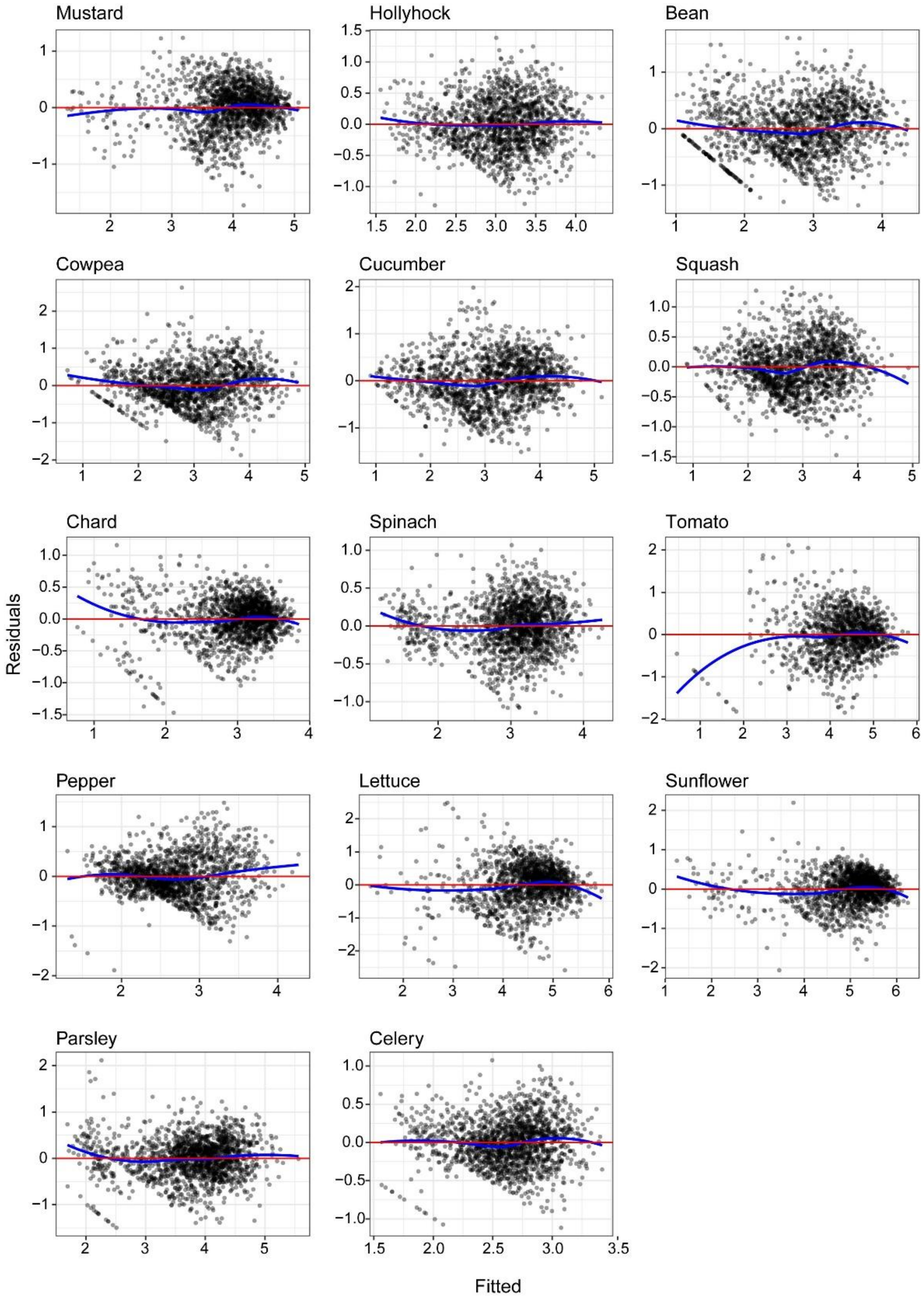

Figure S5. Residual versus fitted plots from the single species linear mixed models fit to log transformed raw lesion data. Scatter plots display the relationship between the fitted values (predicted lesion area in mm²) and the residuals for each of the host species analyzed. Dots represent individual residuals for each biological replicate, while the blue solid line represents a LOESS smoothing curve illustrating the trend of residual means.

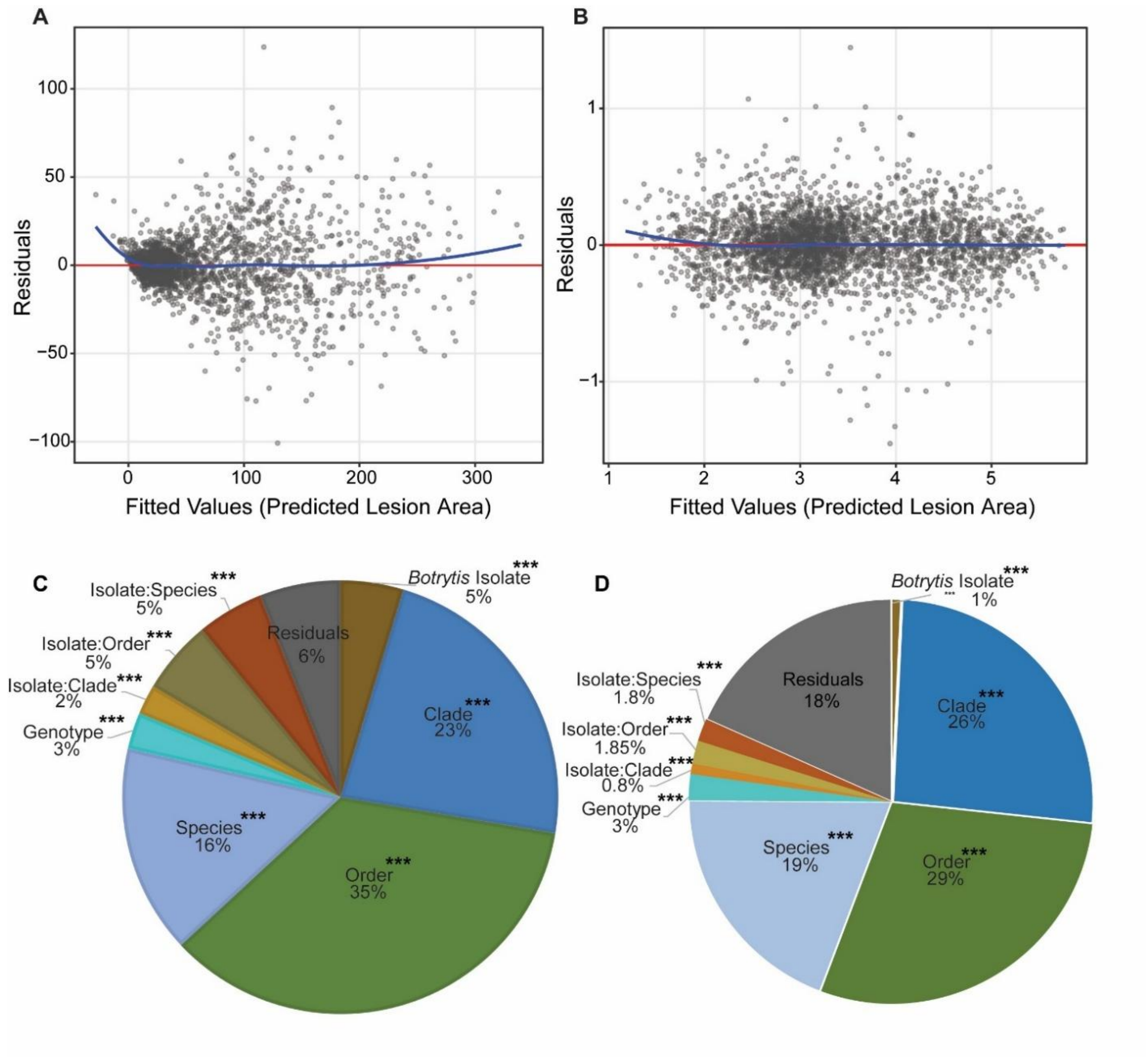

Figure S6. Residual diagnostics and variance partitioning from the multi-host linear mixed model. Scatter plots display the relationship between the fitted values (predicted lesion area in mm²) and the residuals for the multi host linear mixed model **(A)** fitted to LsMeans obtained from raw lesion data **(B)** fitted to LsMeans obtained from log transformed lesion data. Each point represents individual residuals for each biological replicate, while the blue solid line represents a LOESS smoothing curve illustrating the trend of residual means. **(C)** Variance partitioning from the multi-host two-stage linear model fitted to LsMeans obtained from raw lesion data. **(D)** Variance partitioning obtained using the random effect subtraction approach. In this approach, random intercepts for Experiment, Tray, and Plant were first estimated from the full mixed model

and subtracted from the response. The adjusted response was then analyzed using a fixed effect model with the same hierarchical phylogenetic structure.Both the pie charts quantify the proportion of total variance explained by host phylogenetic levels (clade, order, species, genotype), *B. cinerea* isolate, and their interactions. Host levels were modeled hierarchically as Clade > Order > Species > Genotype. For visual clarity, nested terms are simplified in the figure: Genotype represents Clade:Order:Species:Genotype, and Species represents Clade:Order:Species. Interaction terms follow a similar abbreviated convention (e.g., Isolate:Species). Asterisks indicate components with $p < 0.001$.

The variance proportions for the host levels from the subtraction framework closely match those from the two-stage model. This consistency confirms that the dominant source of variation arises from host phylogenetic structure rather than experimental design effects and supports the robustness of the modeling strategy.

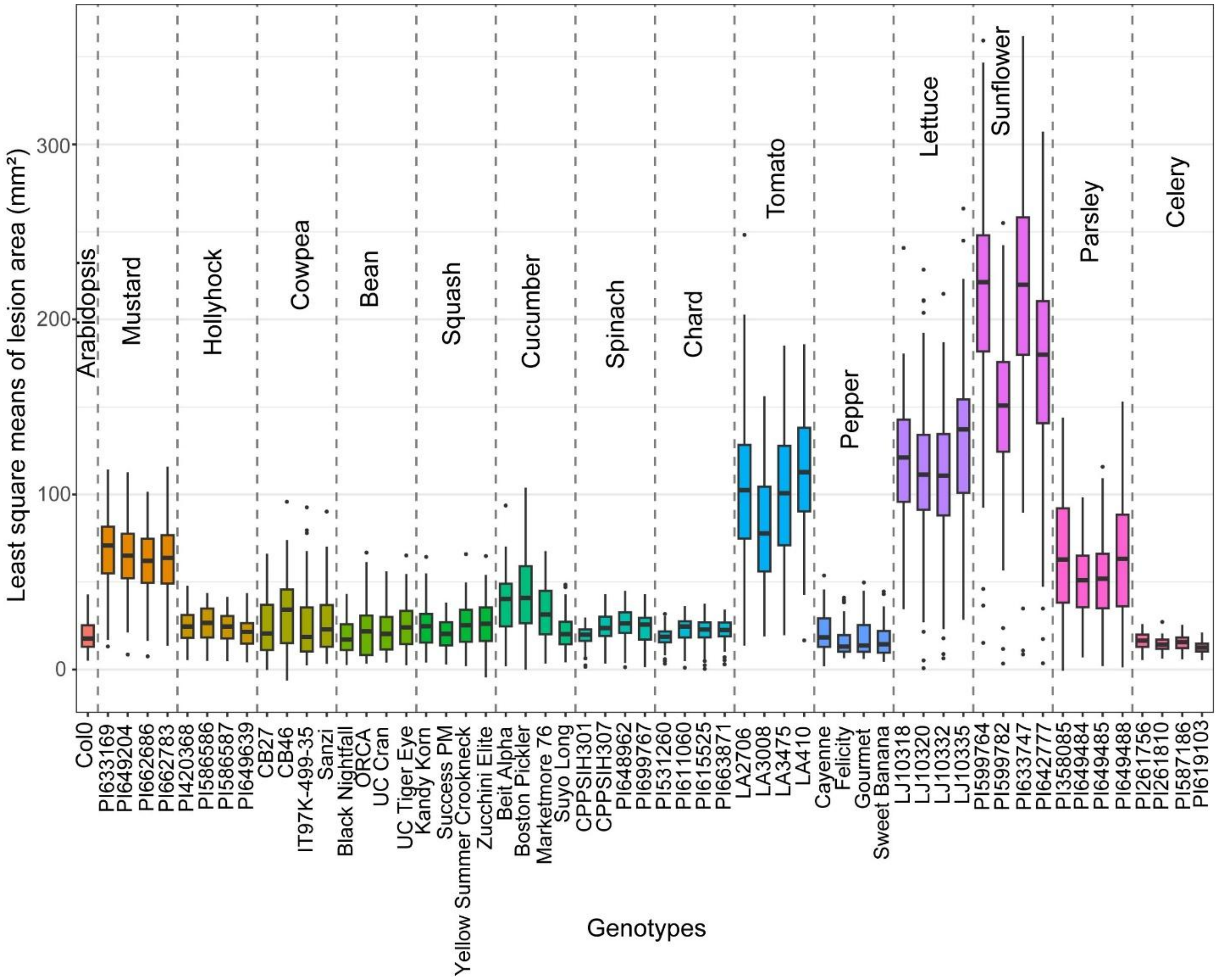

Figure S7. Lesion development and host susceptibility across eudicot species. (B) Boxplots showing least squares mean (LS-mean) lesion area (mm²) at 72 hpi for individual genotypes across 15 eudicot plant species. Genotypes are grouped by species and separated by dotted lines. The species name is labeled once per group above the corresponding set of boxplots, and genotype names are shown along the x-axis. Species are arranged according to their phylogenetic order and clade classification. Arabidopsis is represented by a single genotype (Col-0), while all other species are represented by four genotypes each.

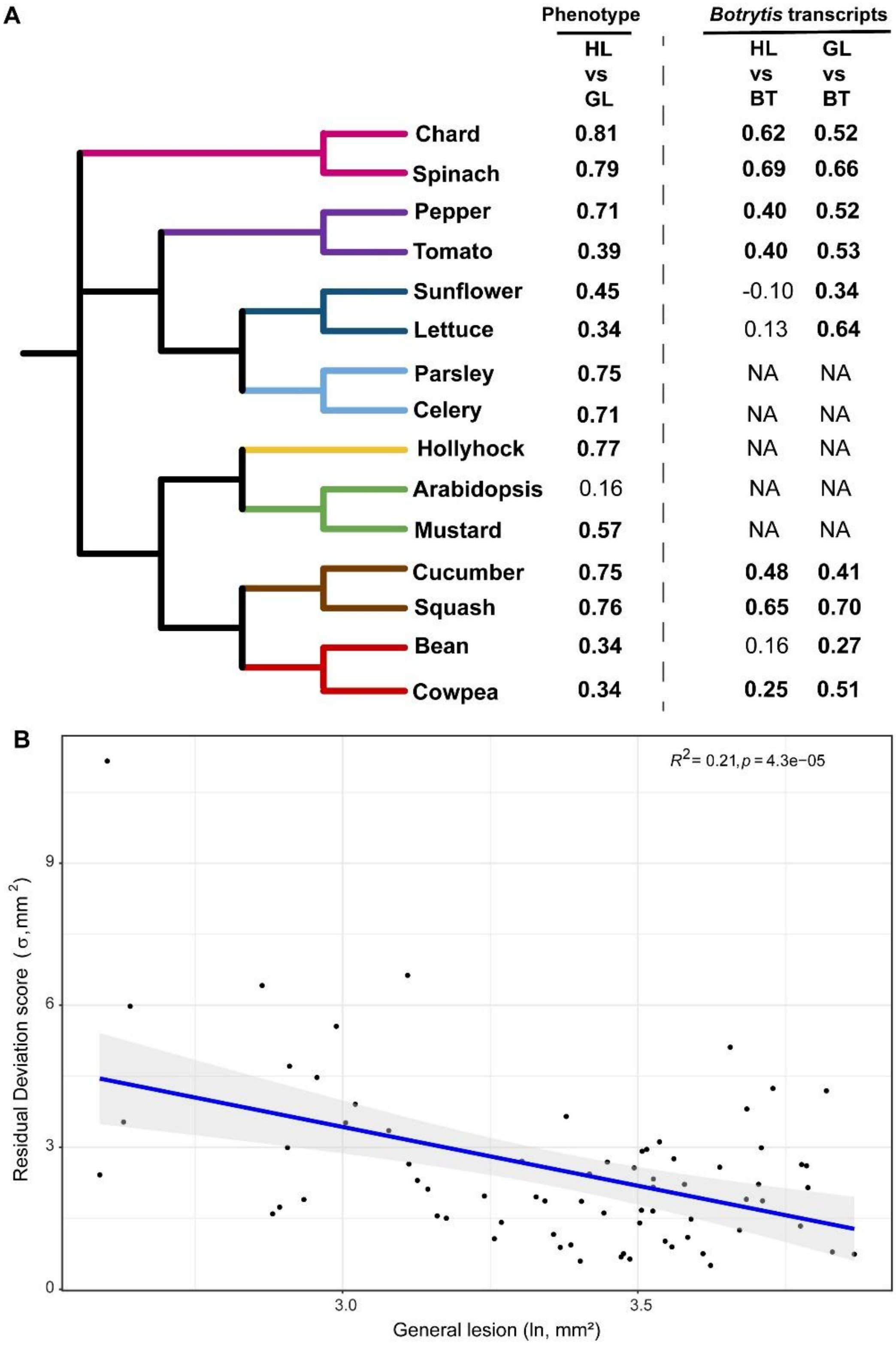

Figure S8: Phylogenetic patterns and descriptors of lesion size variation in *Botrytis cinerea* infections across eudicot hosts. (A) Phylogenetic tree of 15 eudicot plant species used in *B.*

cinerea infection assays, color-coded by plant order. The adjacent table summarizes the Pearson correlation (r) values evaluating the association of different variables with lesion size outcomes. The GL (general lesion, ln) variable represents the mean lesion size across all hosts for each isolate. This was recalculated independently for each species to exclude the focal species and avoid overestimation. HL (host-dependent lesion, ln) for each isolate was calculated as the mean lesion on a given host, while correcting for the genotype-level variation. BT (*B. cinerea* transcript abundance) represents total *B. cinerea* transcript abundance at 48 hpi (a proxy for fungal biomass). "NA" indicates species for which transcriptomics was not performed. Bold values indicate statistically significant predictors ($p < 0.05$). Full plots are shown in **Figures S9, S11, and S12**. **(B)** Relationship across isolates between the residual standard error (σ) value and general lesion estimates. For each isolate, sigma was calculated from the regression of host dependent lesion on general lesion across species. Sigma represents the square root of the mean squared residual adjusted for degrees of freedom, providing a single descriptor of host range variance. Higher sigma values indicate greater deviation from the general lesion trend.

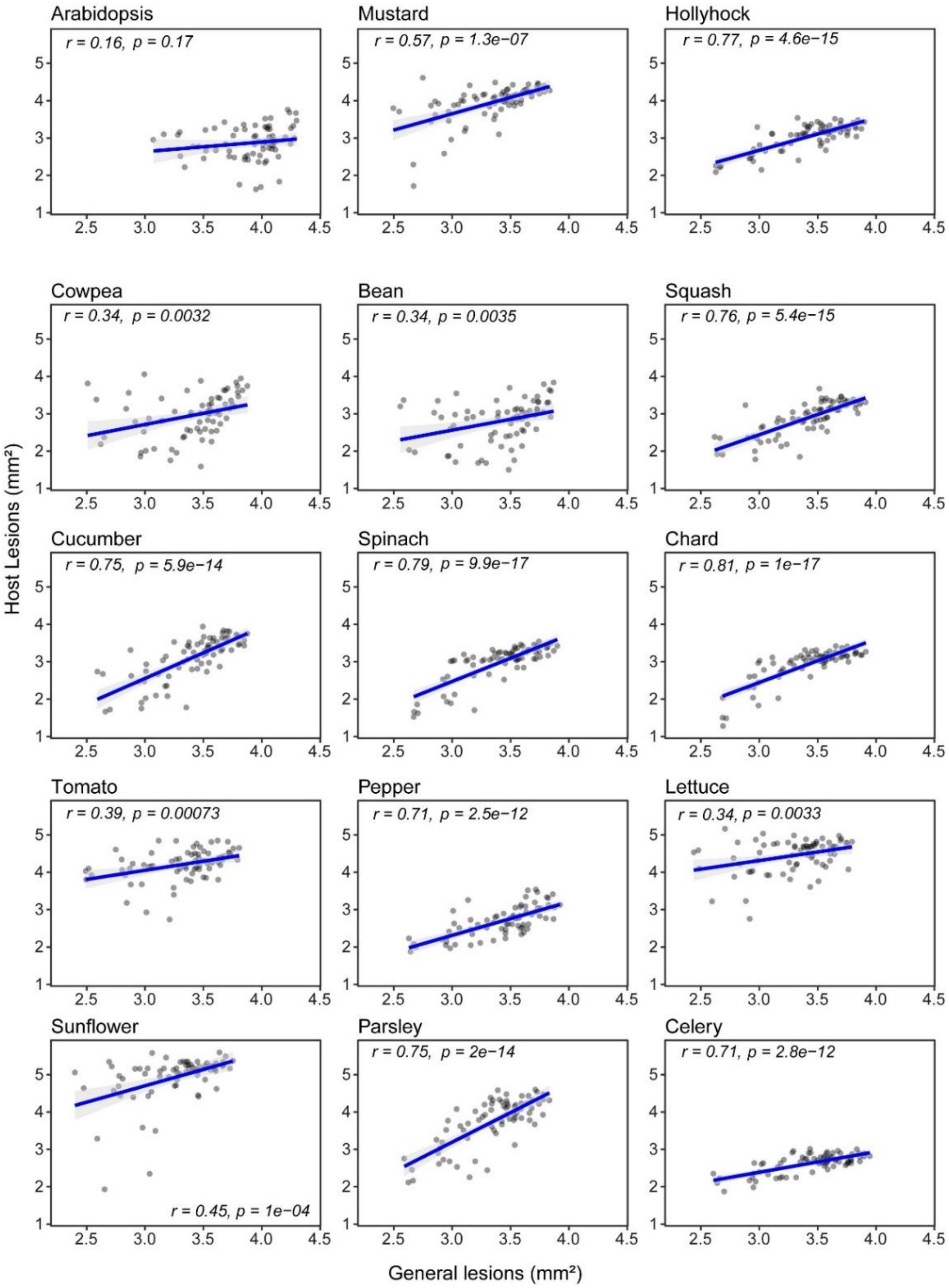

Figure S9. Correlation between general lesion and host lesion size across 15 eudicot hosts. Scatterplots illustrate the relationship between log transformed general lesion size (x-axis; average lesion size across all hosts, excluding the focal host) versus log transformed host-specific lesion size (y-axis; lesion size measured on the focal host) for each of the 15 eudicot plant species. Each point represents a single *B. cinerea* isolate. The strength and significance of the association were assessed using Pearson correlation; correlation coefficients (r) and *p*-values are provided within each panel.

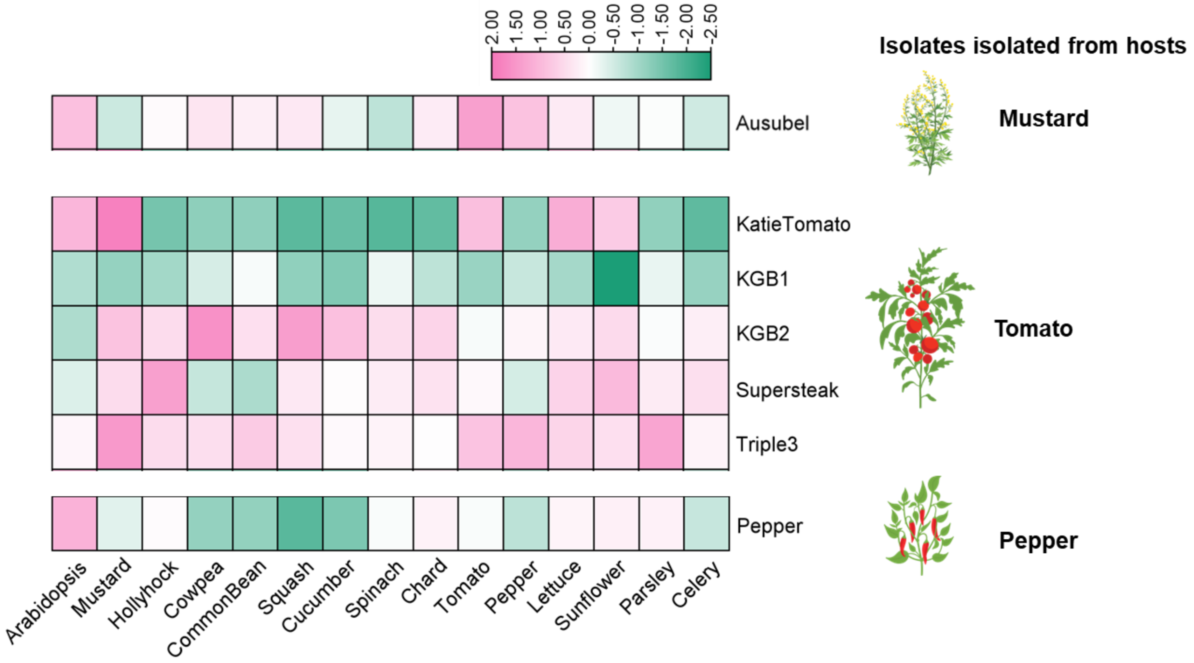

Figure S10. Source-host specialization patterns of *B. cinerea* isolates. Z-scaled heatmap showing lesion size across 15 eudicot plant hosts for seven *B. cinerea* isolates, each originating from a specific host. Isolates include five tomato-derived isolates (*KatieTomato*, *KGB1*, *KGB2*, *Supersteak*, and *Triple3*), one mustard-derived isolate (*Ausubel*), and one pepper-derived isolate (*Pepper*). Z-scaling was calculated by subtracting the mean lesion size of each isolate across all hosts and dividing by the standard deviation. Positive values indicate lesions larger than the isolate's average across hosts. Negative values indicate lesions smaller than the isolate's average. Rows represent individual *B. cinerea* isolates, and columns represent host plant species. Pink indicates higher-than-average values and green indicates lower-than-average values.

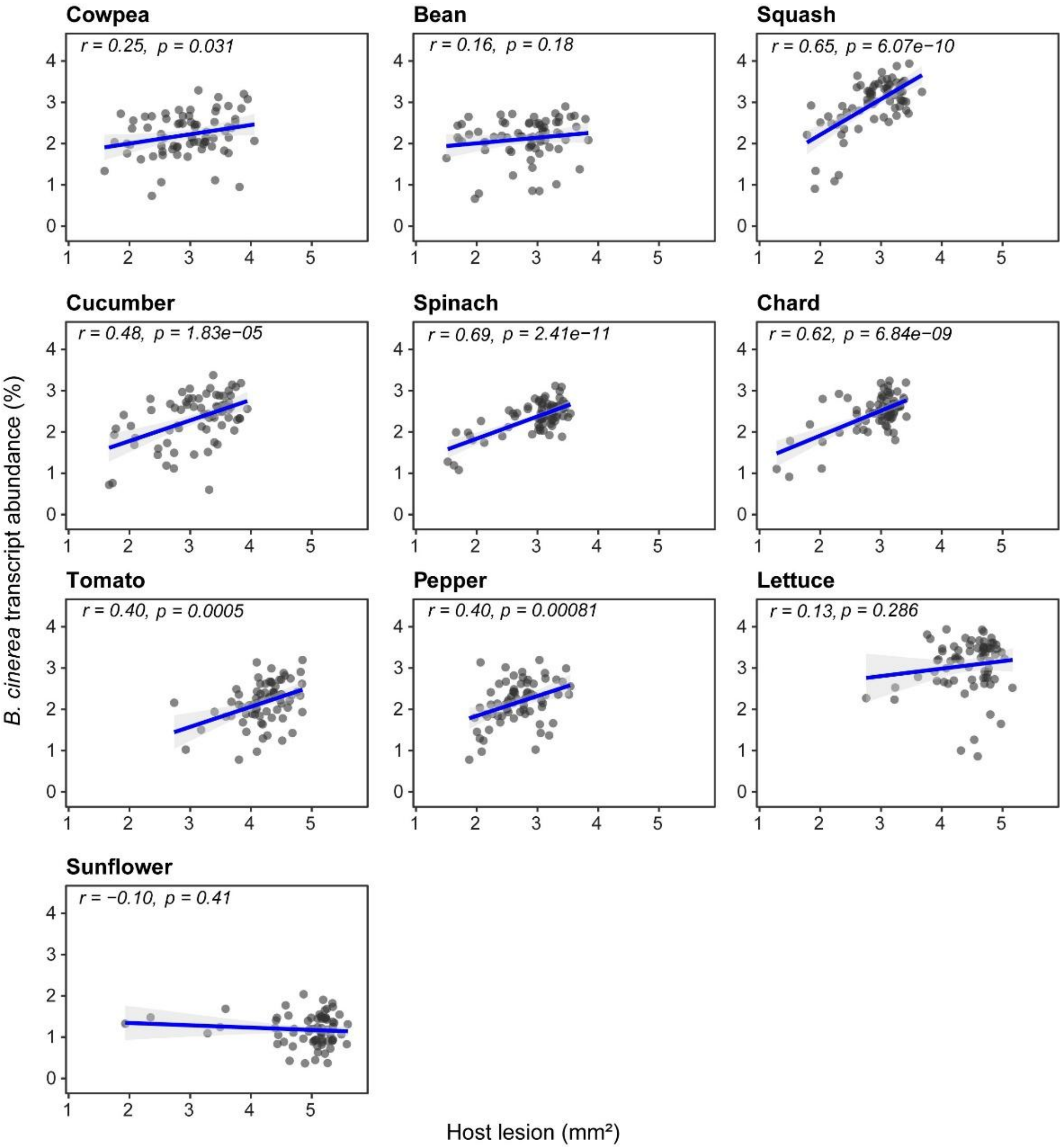

Figure S11. Correlation between *B. cinerea* transcript abundance and host lesion across 10 eudicot hosts. Scatterplots show the relationship between log transformed lesion size at 72 hpi on each host species (host lesion; x-axis) and early *B. cinerea* transcript abundance at 48 hpi (y-axis; log transformed). Each point represents a single *B. cinerea* isolate. The strength and significance of the association were assessed using Pearson correlation; correlation coefficients (r) and *p*-values are provided within each panel.

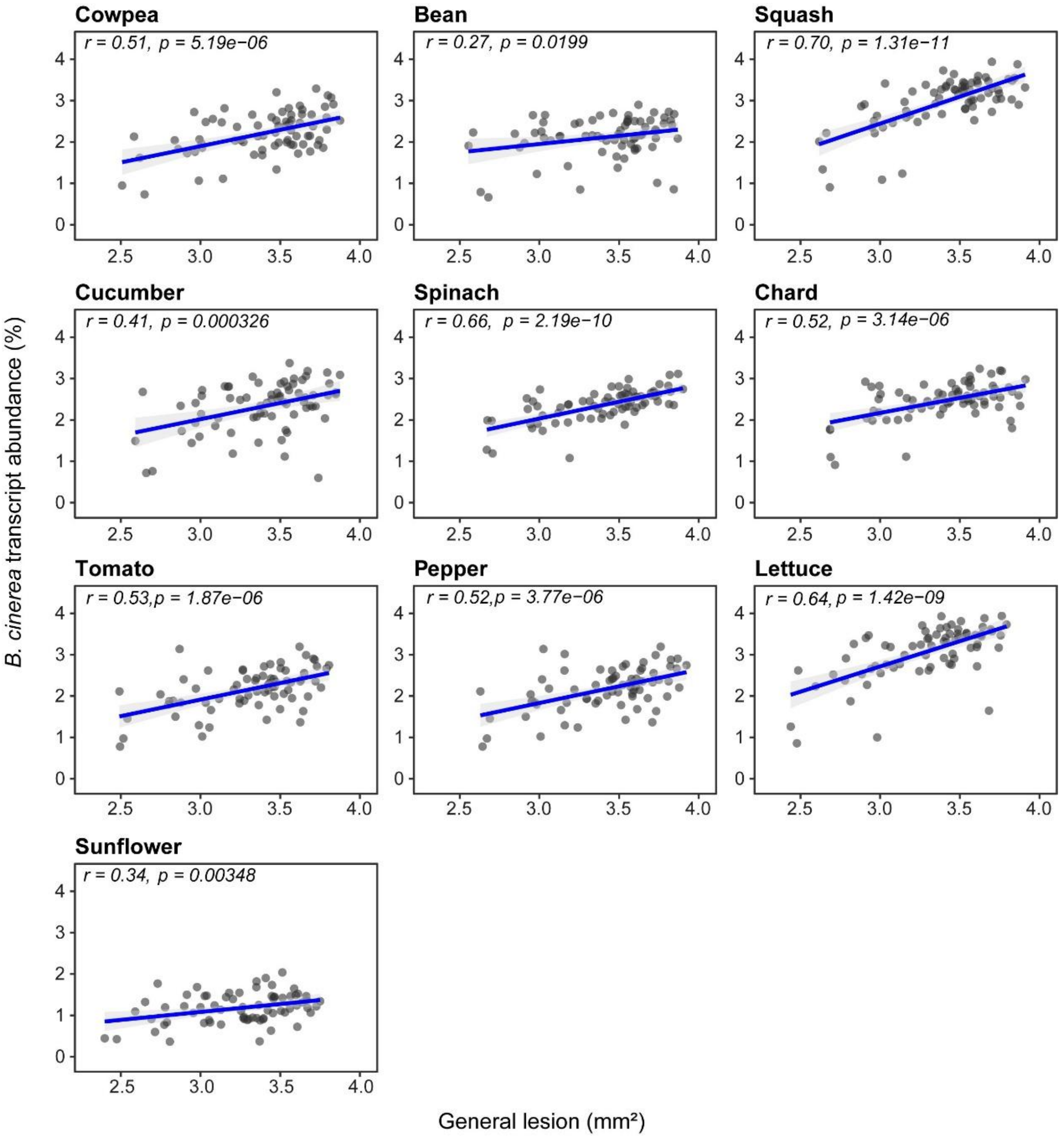

Figure S12. Correlation between *B. cinerea* transcript abundance and general lesion across 10 eudicot hosts. Scatterplots show the relationship between log transformed general lesion at 72 hpi (mean lesion area across all host species; x-axis) and early *B. cinerea* transcript abundance at 48 hpi (y-axis; log transformed) for each host species. Each point represents a single *B. cinerea* isolate. The strength and significance of the association were assessed using Pearson correlation; correlation coefficients (r) and *p*-values are provided within each panel.

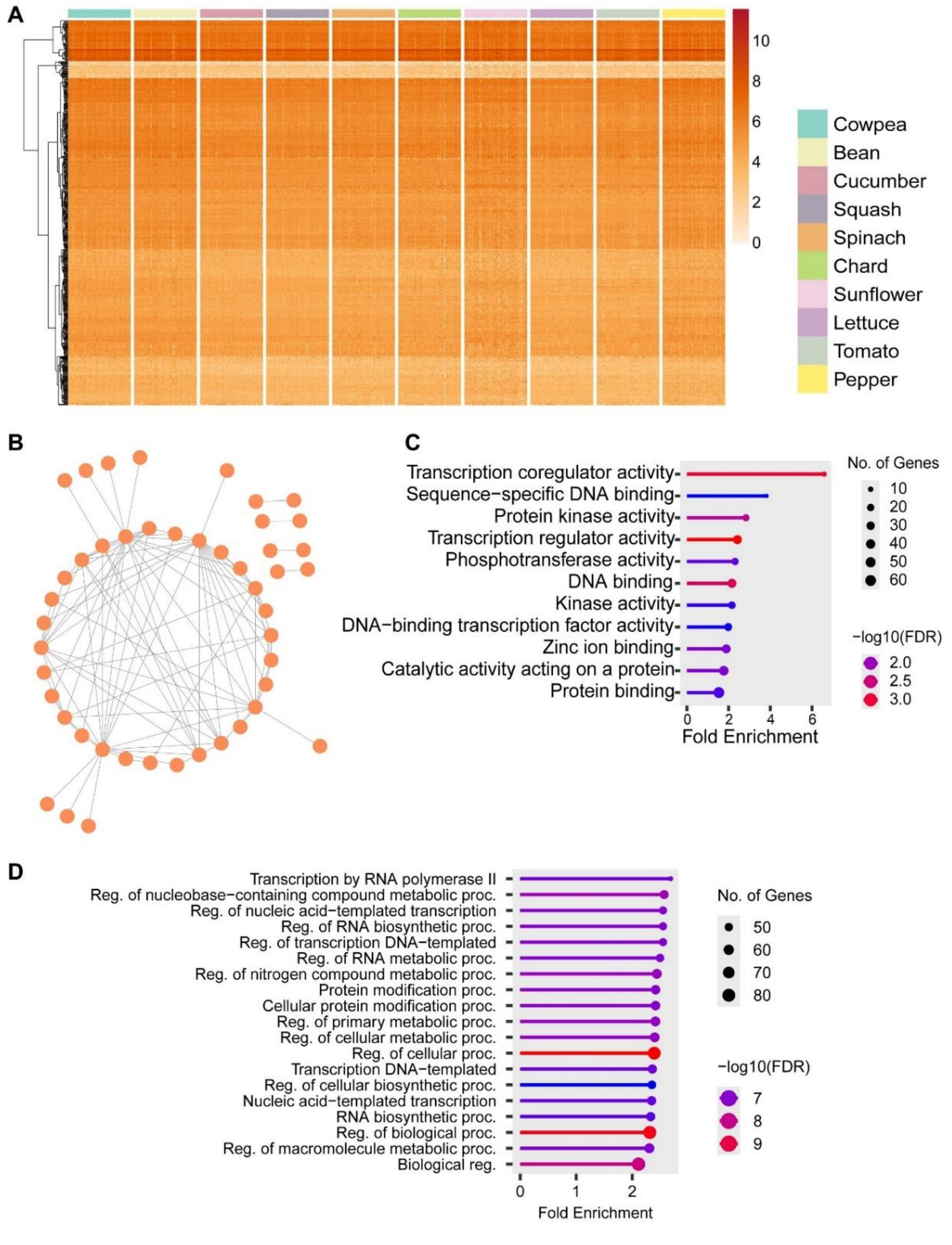

Figure S13. Expression patterns, co-expression network, and functional enrichment of 500 low-entropy *B. cinerea* genes. (A) Heatmap showing $\log_2$ (CPM + 1) expression levels of low-entropy genes across 72 *B. cinerea* isolates infecting 10 eudicot plant hosts. Columns represent individual *B. cinerea* isolates, grouped by host species (color-coded) and arranged according to plant phylogeny. Rows represent individual *B. cinerea* genes hierarchically clustered by expression

profile (Euclidean distance, complete linkage). Normalized CPM values were transformed to $\log_2(CPM + 1)$ for visualization only, to improve dynamic range in the color scale while preserving relative expression patterns. No Z-score scaling was applied to preserve absolute expression differences; the heatmap color scale represents increasing expression intensity from low (light) to high (dark). **(B)** Co-expression network identified by testing the top 500 conserved genes. The network includes 46 genes and was constructed from pairwise Pearson correlation analysis using the absolute correlation coefficient ($|r| \geq 0.7$) across the entire *B. cinerea* transcriptome dataset (72 isolates × 10 hosts = 720 samples). Nodes represent individual genes; edges represent significant co-expression relationships. **(C–D)** Gene Ontology (GO) enrichment analysis for the 500 low-entropy genes. Dot plots show enriched **(C)** molecular function and **(D)** biological process categories, with dot size representing the number of genes per term and color indicating significance level ($-\log_{10}$[FDR]), generated using ShinyGO.

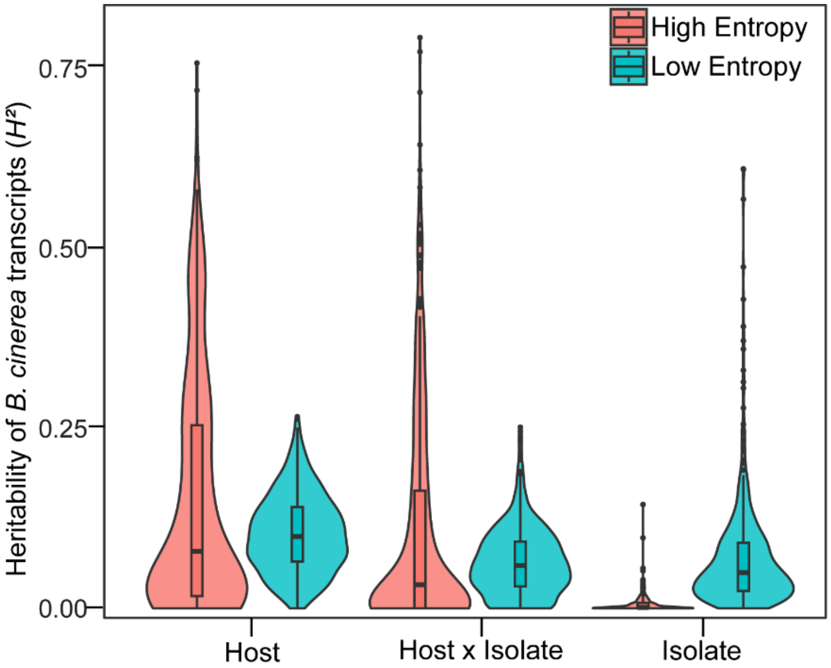

Figure S14. Distribution of broad-sense heritability (*H^2*) of *B. cinerea* transcripts explained by isolate, host species, and their interaction for high- and low-entropy genes. Violin and box plots show the distribution of H^2 for *B. cinerea* gene expression across 72 isolates infecting 10 eudicot host species. Heritability estimates were partitioned into three components based on linear mixed-effects models: isolate: genetic variation attributable to 72 *B. cinerea* isolates; host: variation attributable to 10 eudicot host plant species, and their respective interactions.

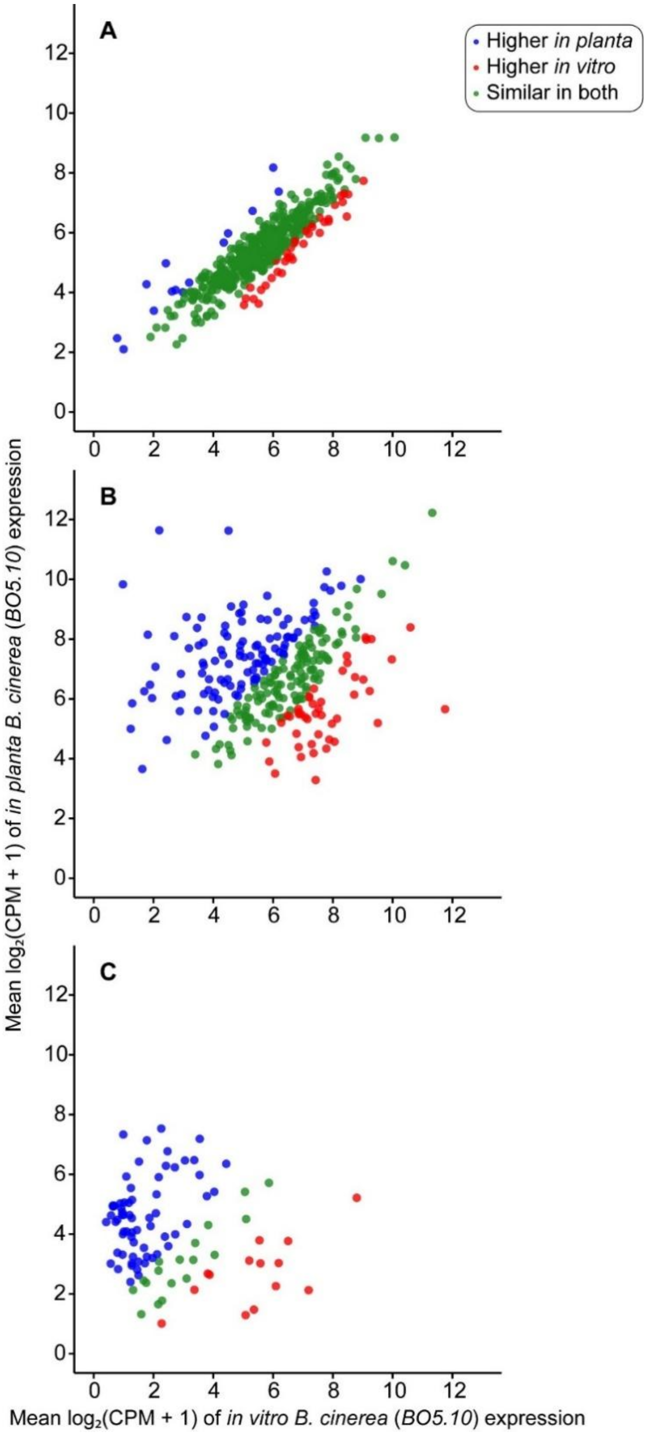

Figure S15. Comparison of *B. cinerea* (*BO5.10* isolate) transcript accumulation *in planta* and *in vitro*. Scatterplots show average expression ($\log_2$ [CPM + 1]) of *B. cinerea* genes measured *in planta* (across 10 eudicot hosts) versus *in vitro* (PDB media) for the *B05.10* isolate. Each point represents a single gene, colored by relative expression bias: **(A)** 500 low-entropy (conserved) genes. **(B)** 287 general lesion-associated genes. **(C)** 434 high-entropy (host-specific) genes; only 97 showed detectable expression *in vitro*.

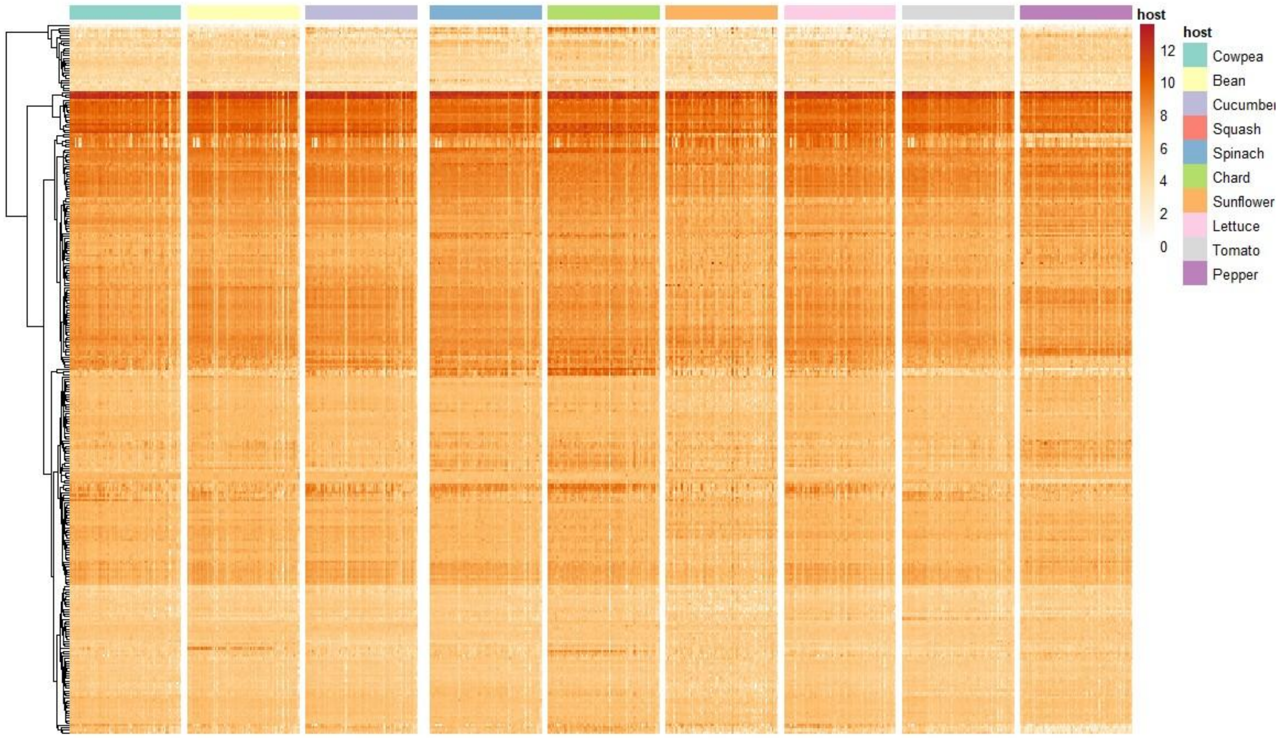

Figure S16. Expression patterns of general lesion–associated *B. cinerea* transcripts across 10 eudicot species. Heatmap of $\log_2$ (CPM + 1) expression values for the same 287 genes across all 72 *B. cinerea* isolates infecting 10 eudicot species. Columns represent individual *B. cinerea* isolates. Isolates are ordered from higher to lower general lesion potential and grouped by host species. Host groups are color coded and arranged according to host phylogenetic relationships. Rows represent individual *B. cinerea* genes hierarchically clustered by expression profile (Euclidean distance, complete linkage). Normalized CPM values were transformed to $\log_2$(CPM + 1) for visualization only, to improve dynamic range in the color scale while preserving relative expression patterns. No Z-score scaling was applied to preserve absolute expression differences; the heatmap color scale represents increasing expression intensity from low (light) to high (dark).

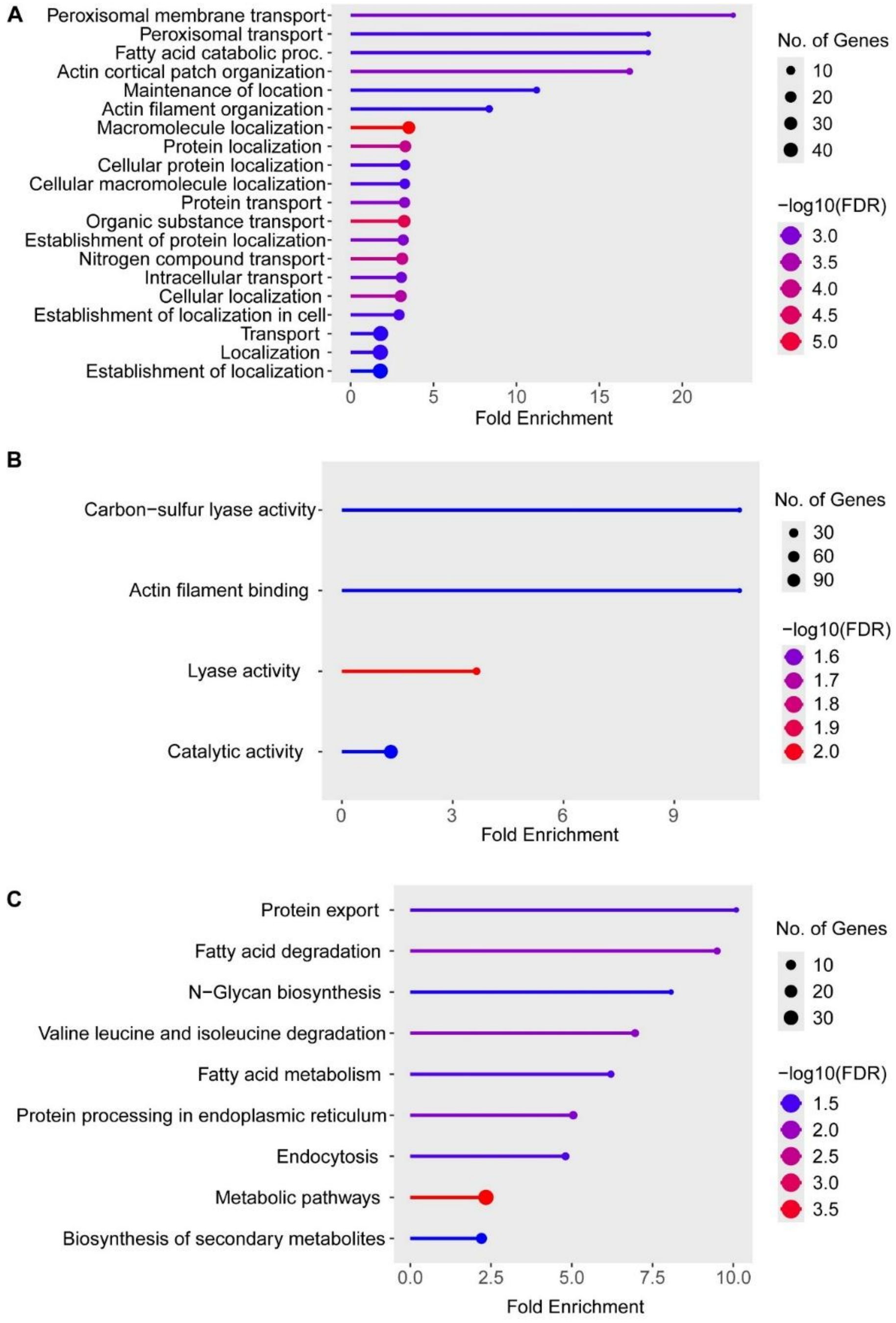

Figure S17. Functional enrichment analysis of general lesion-associated *B. cinerea* genes. Gene Ontology (GO) and KEGG enrichment analyses were performed for 287 *B. cinerea* transcripts significantly associated with general lesion size using Shiny GO. Significantly enriched categories are shown for **(A)** GO Biological Processes (BP), **(B)** GO Molecular Functions (MF), and **(C)** KEGG pathways. Dot plots display enriched terms, with dot size corresponding to the number of genes annotated in each category and dot color representing statistical significance ($-\log_{10}$(FDR)).

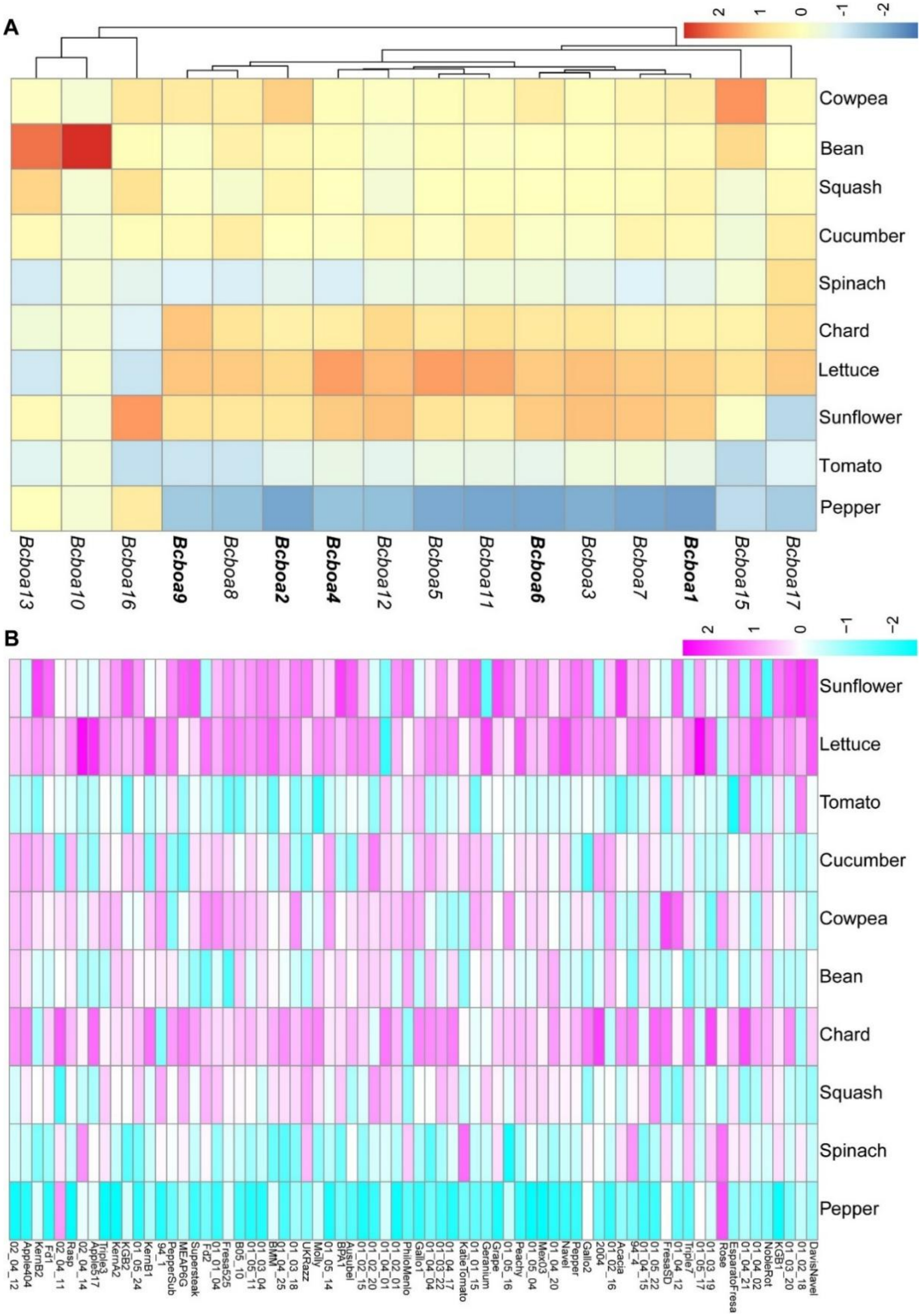

Figure S18. Expression patterns of *B. cinerea Botcinic acid* (*BOA*) cluster genes across 10 eudicot species. (A) Heatmap showing z-scaled (column) expression of individual BOA cluster

genes (columns; *Bcin01g00010*- *Bcin01g00160*) averaged across all isolates infecting each host species (rows). Genes included are based on the full annotated BOA biosynthetic cluster. Hierarchical clustering was applied to genes and host species are arranged as per phylogeny. Genes highlighted in bold (*Bcboa1*, *Bcboa2*, *Bcboa4*, *Bcboa6*, *Bcboa9*) are members of a co-expressed submodule identified in general lesion-associated network analysis. **(B)** Heatmap showing z-scaled (column) expression of the BOA cluster averaged at the gene-set level for each isolate-host combination. Columns represent individual *B. cinerea* isolates, and rows represent host species. Isolates are ordered from left to right based on general lesion size, ranging from isolate 02_04_12 (highest average lesion) to Davis Navel (lowest). Host species (rows) are arranged by susceptibility (mean species-level lesion size), from Pepper (lowest susceptibility) to Sunflower (highest).

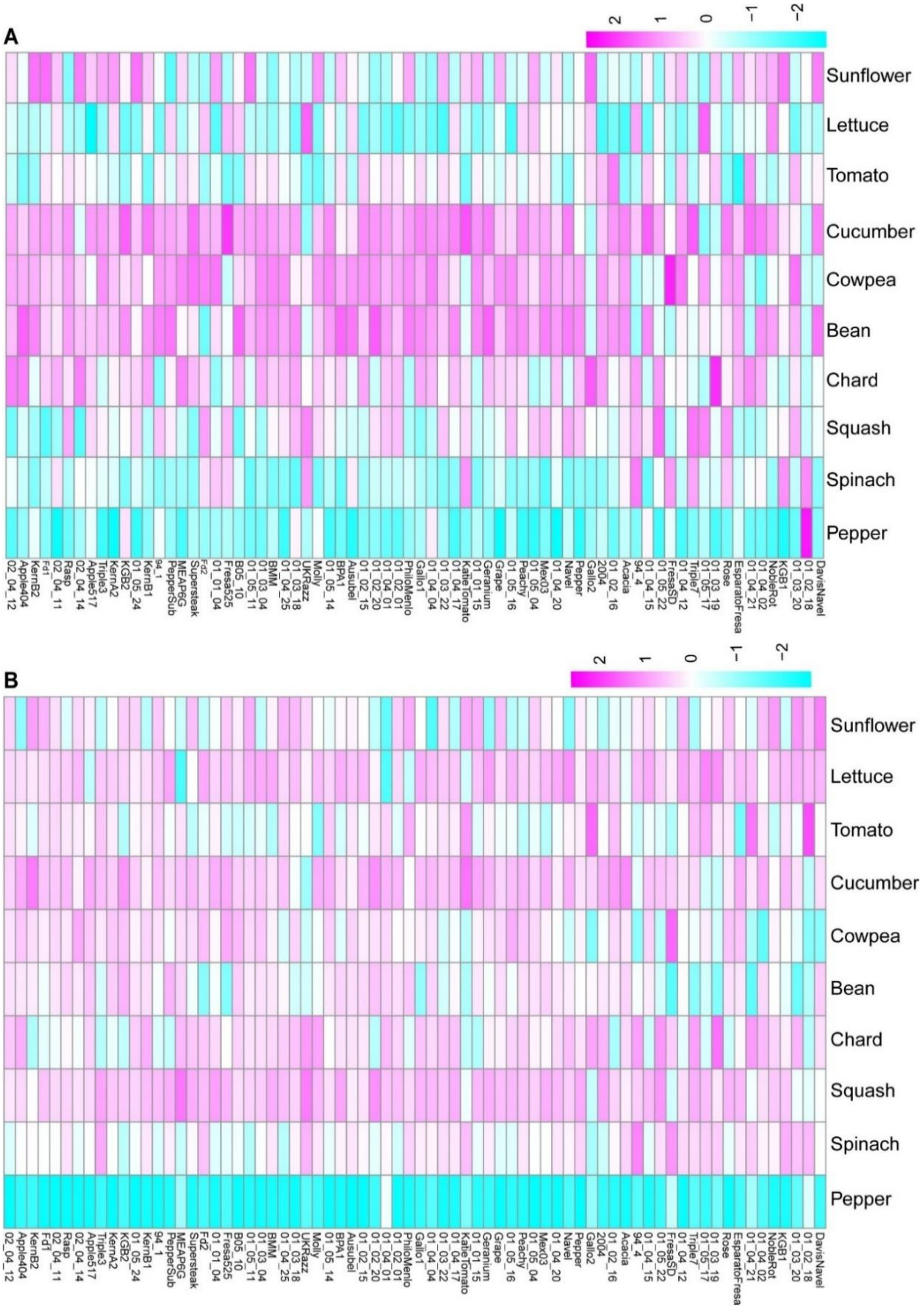

Figure S19. Expression patterns of canonical virulence genes across 72 *B. cinerea* isolates infecting 10 eudicot host species. (A) Z-scaled heatmap (column) showing expression of all genes in the *Botrydial* (*BOT*; *Bcin12g06370–Bcin12g06410*) biosynthetic cluster, averaged at the gene-

set level across isolate-host combinations. **(B)** Z-scaled heatmap (column) showing expression of the polygalacturonase gene *Bcpg1* (*Bcin14g00850*) across the same isolate-host matrix. Columns represent individual isolates; rows are grouped by host species. Isolates are ordered from left to right based on their general lesion values, with isolate *02_04_12* showing the highest general lesion and isolate *Davis Navel* showing one of the lowest. Host species are arranged from least to most susceptible based on their species level lesion means, with Pepper showing the lowest susceptibility and Sunflower the highest.

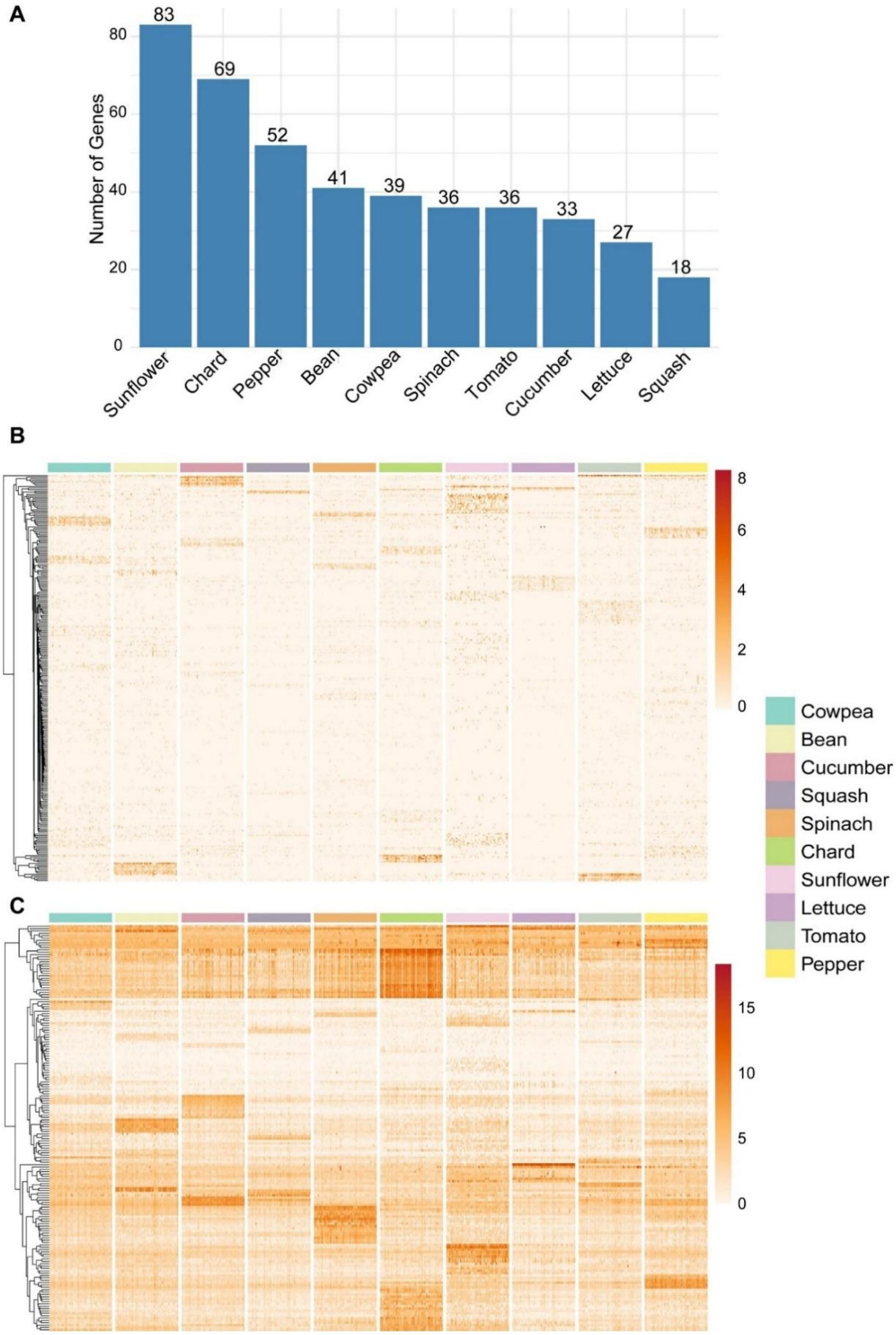

Figure S20. Distribution and expression patterns of single host–specific genes across *B. cinerea* isolates infecting 10 eudicot host species. (A) A gene was defined as single host-specific

if its expression in one host was ≥ 1 standard deviation (SD) above its mean expression across all hosts. Each gene was assigned to the host in which it showed ≥1 SD higher expression. The bar plot shows the number of single host-specific genes identified per host. **(B-C)** Heatmap of log_2 (CPM + 1) expression values for single-host-specific genes, shown across all 72 *B. cinerea* isolates with (**B)** 262 genes expressed in only a few hosts while **(C)** 172 genes expressed across hosts, but with expression ≥ 1 SD in one host compared to others. Columns represent individual *B. cinerea* isolates, grouped by host species (color-coded) and arranged according to plant phylogeny. Rows represent individual *B. cinerea* genes hierarchically clustered by expression profile (Euclidean distance, complete linkage). In all panels, normalized CPM values were transformed to log_2 (CPM + 1) for visualization only, to improve dynamic range while preserving relative expression patterns. No Z-score scaling was applied to preserve absolute expression differences; the heatmap color scale represents increasing expression intensity from low (light) to high (dark).

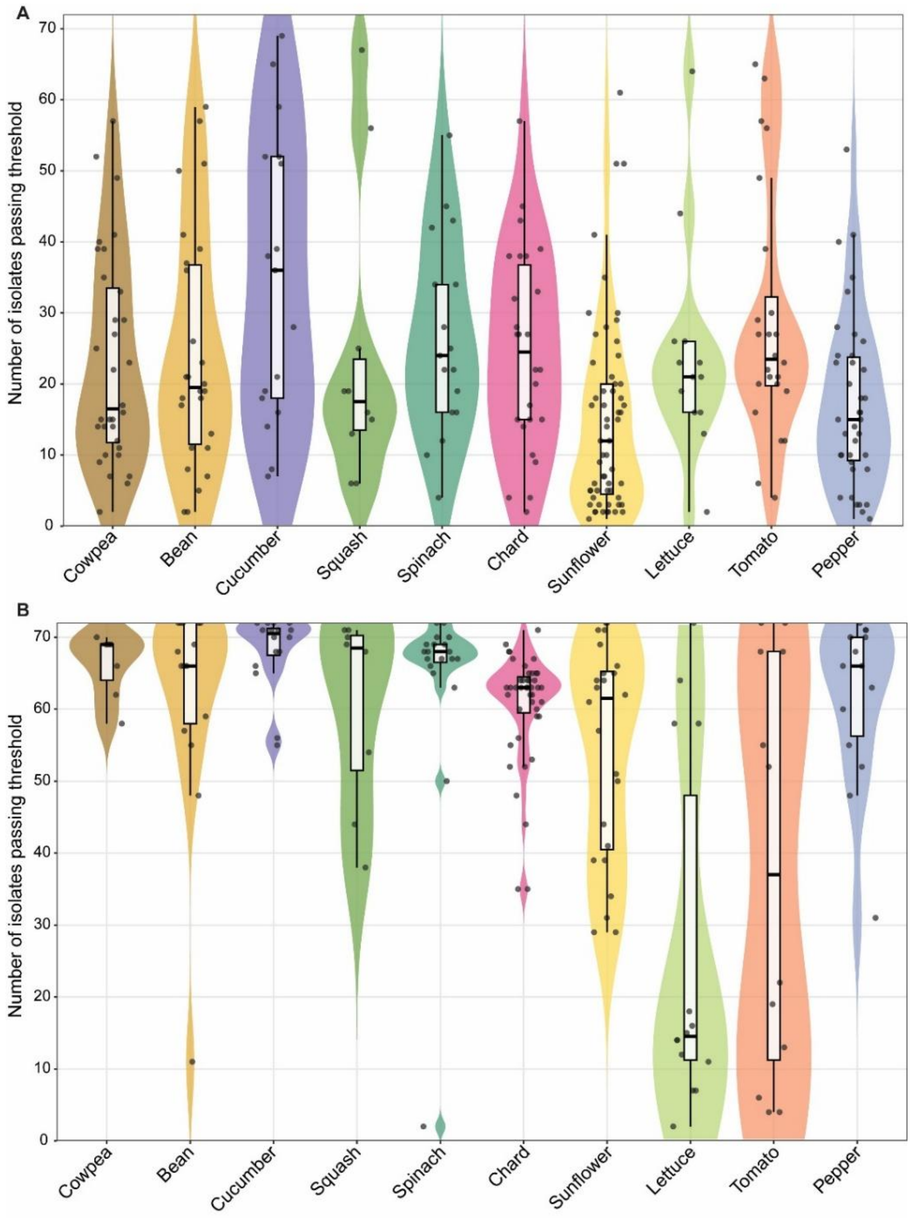

Figure S21. Percentage of isolates passing the high-expression threshold for host-specific genes. Genes were classified as host-specific if its average expression in one host was at least one standard deviation above its mean expression across all hosts. Each gene was then assigned to the host in which it exceeded this threshold. For each host-specific gene, the percentage of isolates expressing the gene above the high-expression threshold (exceeding one SD threshold) was determined. The host-specific genes were then parsed into those **(A)** Genes largely un-expressed except within the specific host (262 genes). This group shows high population-level variability (average 34% of isolates passing threshold). **(B)** Genes with low background expression across all hosts but elevated expression in the specific host (172 genes). This group shows higher population-level conservation (average 78% of isolates passing threshold), though the degree of conservation varies by host. Each dot represents an individual gene.

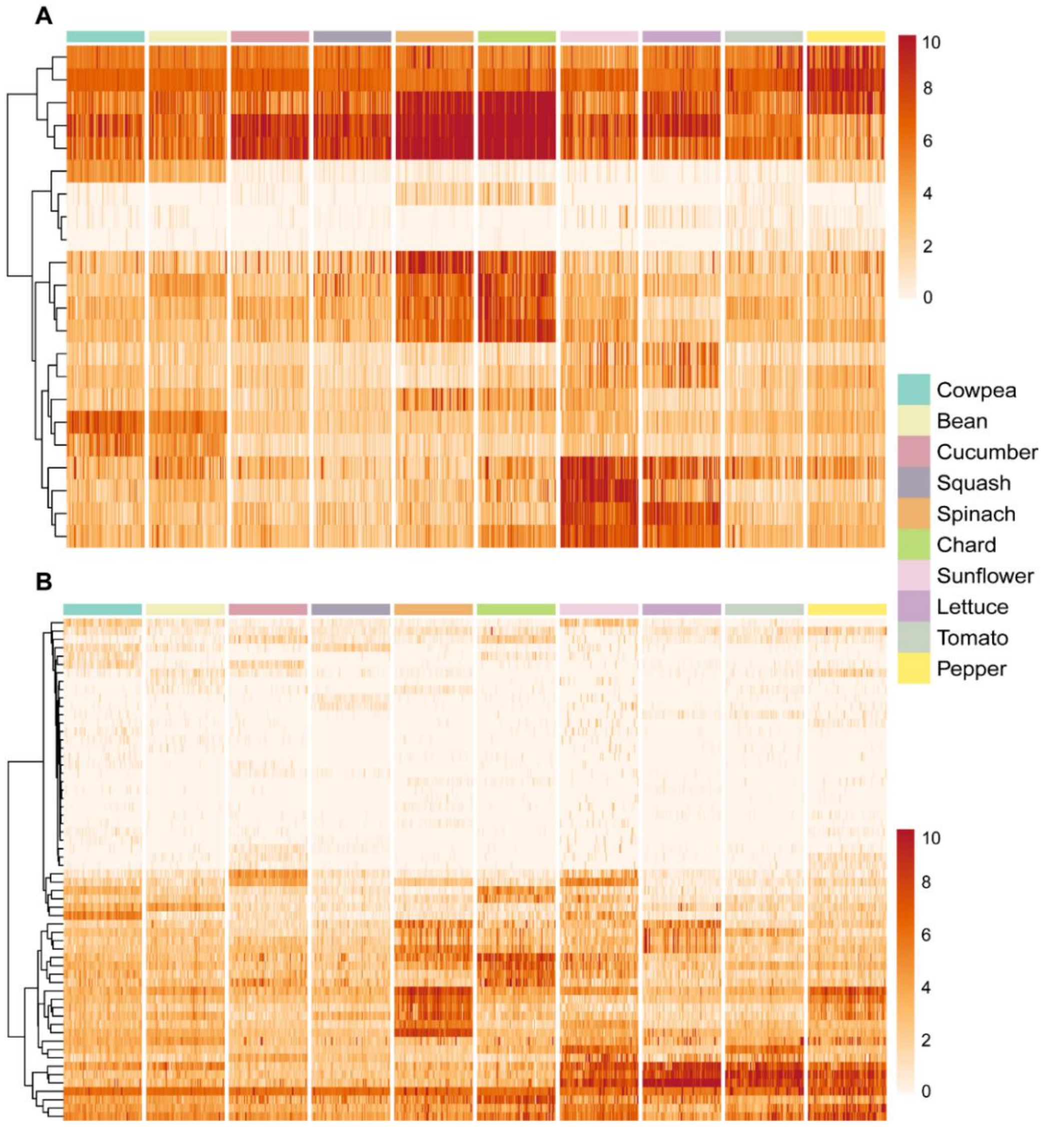

Figure S22. Multi-host-specific gene expression profiles across *B. cinerea* isolates infecting 10 eudicot hosts. (A-B) Heatmaps of $\log_2$ (CPM + 1) expression values for genes showing moderate host specificity, defined as having expression ≥1 standard deviation above the gene's mean in only two or three host species. All 72 *B. cinerea* isolates are shown across all 10 host. **(A)** Genes (n=22) with high expression in phylogenetically related hosts, suggesting order-specific regulatory responses. **(C)** Genes (n=60) with high expression in two or three phylogenetically unrelated hosts. Columns represent individual *B. cinerea* isolates, grouped by host species (color-coded) and arranged according to plant phylogeny. Rows represent individual *B. cinerea* genes hierarchically clustered by expression profile (Euclidean distance, complete linkage). Normalized CPM values were transformed to $\log_2$(CPM + 1) for visualization only, to improve dynamic range in the color scale while preserving relative expression patterns. In all panels, no Z-score scaling was applied to preserve absolute expression differences; the heatmap color scale represents increasing expression intensity from low (light) to high (dark).

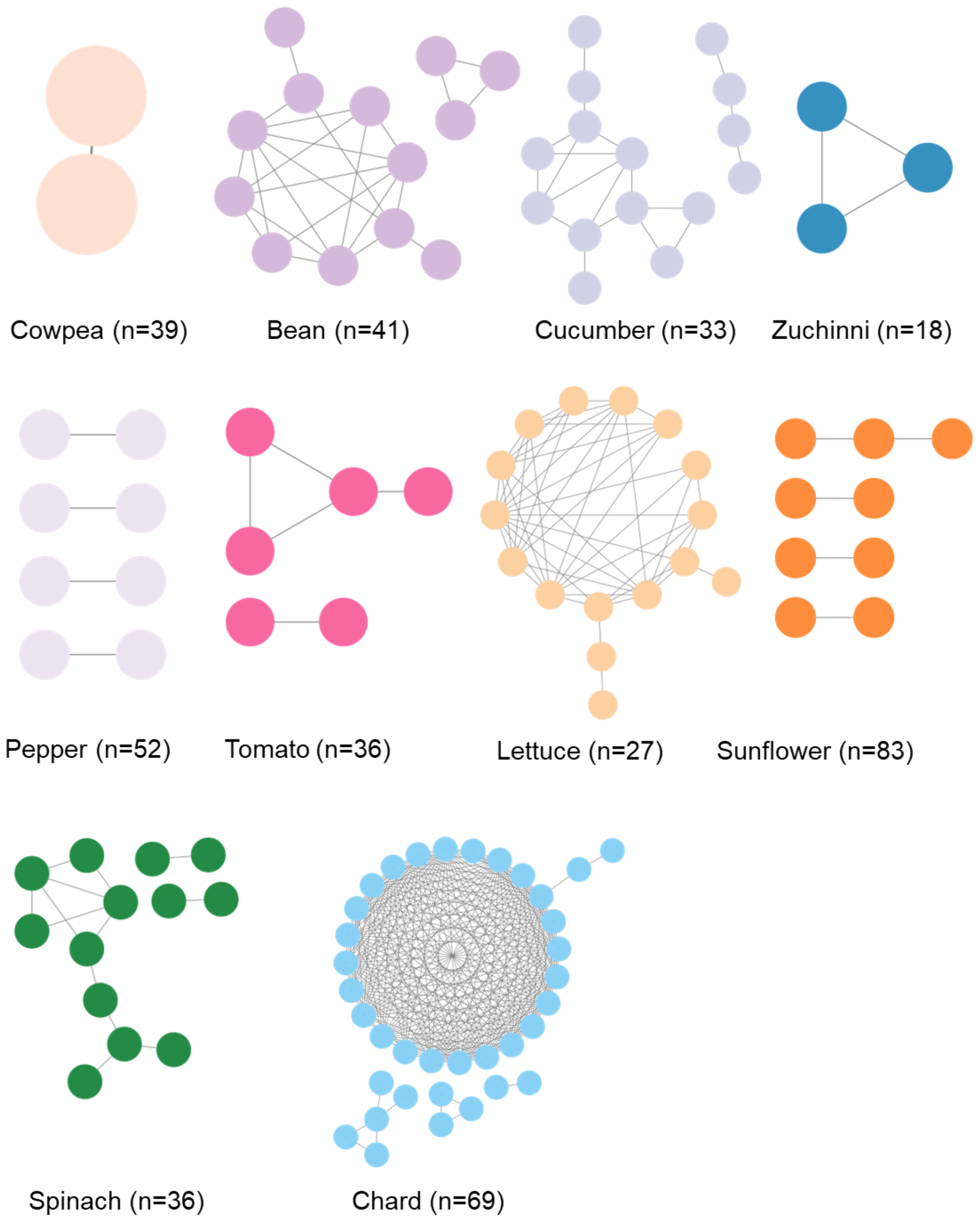

Figure S23. Co-expression networks of *B. cinerea* genes showing host-specific high entropy genes expression in single eudicot hosts. For each host, gene co-expression networks were constructed using Pearson correlation coefficients calculated from gene expression profiles across 72 *B. cinerea* isolates for that host. Nodes represent individual *B. cinerea* genes, and edges denote significant co-expression relationships, defined by the absolute value of the correlation coefficient ($|R| \geq 0.7$). The number of genes (n) shown indicates the total number of host-specific genes used as input for that host.

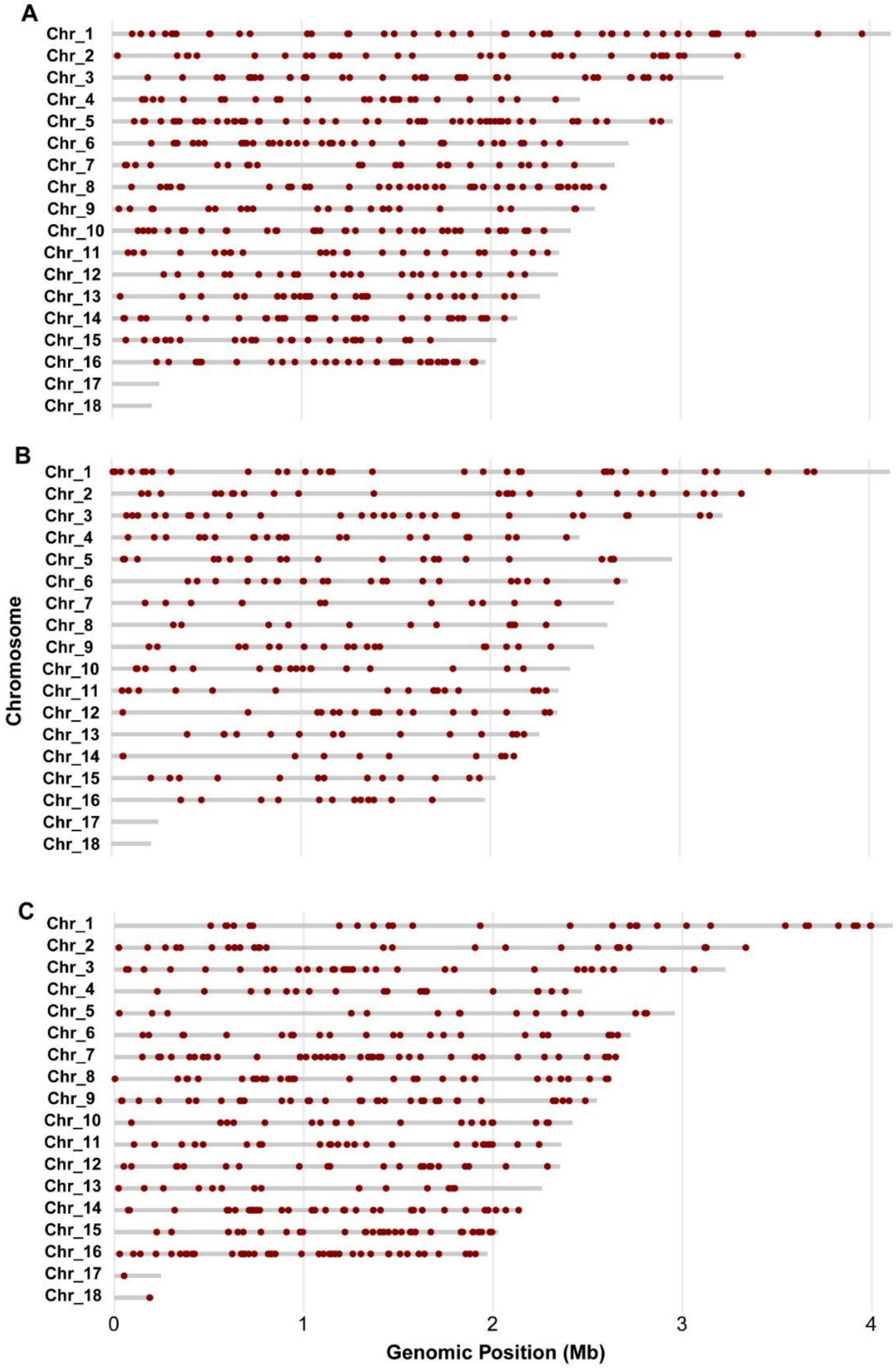

Figure S24. Genomic distribution of entropy-classified and general lesion–associated *B. cinerea* genes across 18 chromosomes. (A) Low-entropy genes. **(B)** General lesion-associated genes. **(C)** High-entropy genes. Plots were generated using ShinyGO. The x-axis indicates chromosomal position in megabase pairs (Mbp); each red dot represents an individual gene.

Supplementary Datasets

Dataset S1: Raw lesion area measurements after filtering out failed lesions. Columns include lesion area, plant genotype, plant ID (the individual plants from where leaves were collected), tray, experiment, isolate identity, and host species.

Dataset S2: Least-square mean lesion areas (mm^2) at 72 hours post-inoculation (hpi) for 15 eudicot species, each with four genotypes.

Dataset S3: Information for 57 genotypes of 15 eudicot species. The germplasm ID corresponds to the germplasm collections (the U.S. Department of Agriculture Germplasm Resources Information Network [USDA GRIN], the Centre for Genetic resources in the Netherland [CGN], UC Davis Tomato Genetic Resource Center [TGRC]), and other IDs represent IDs for species obtained from the commercial seed company. Information on the species and subspecies, common name, order, clade, and cultivar name is provided when available. The germplasm ID/other ID written in bold and underlined was used for RNA sequencing.

Dataset S4: Broad-sense heritability (H^2) estimates for *B. cinerea* gene expression across all hosts ("All"), within plant clades (Rosids, Asterids including Superasterids), and within plant orders (Asterales, Caryophyllales, Fabales, Cucurbitales, Solanales). H^2 values are reported for variance components attributable to *B. cinerea* isolate, host species, and their interaction.

Dataset S5: Shannon entropy values for all *B. cinerea* genes based on host-averaged expression profiles across 10 plant hosts. Genes were categorized into distinct entropy groups, Low, Intermediate, and High, using the Jenks natural breaks classification method.

Dataset S6: List of the 500 lowest-entropy *B. cinerea* genes. For each gene, average $\log_2$(CPM+1) expression across hosts, transcript heritability (H^2), Shannon entropy, and functional annotations are provided.

Dataset S7: Spearman correlation coefficients (ρ) and *p*-values for low-entropy, general lesion-associated, and high-entropy genes with host-dependent lesion for each eudicot species.

Dataset S8: List of 287 general lesion-associated *B. cinerea* genes. For each gene, average $\log_2$(CPM+1) expression across hosts, transcript heritability (H^2), Shannon entropy, and functional annotations are provided.

Dataset S9: List of 434 high-entropy single-host-specific *B. cinerea* genes. For each gene, average $\log_2$(CPM+1) expression across hosts, transcript heritability (H^2), Shannon entropy, and functional annotations are provided. The "Specific to host" column indicates the host(s) to which each gene was assigned based on our z-score criteria.

Dataset S10: List of 82 high-entropy multi-host-specific *B. cinerea* genes. The "Order-specific" column indicates whether gene expression patterns follow host phylogenetic order (i.e., order-aligned) or not. The "Not-specific" label denotes genes whose expression patterns do not align

with host phylogeny. The "Specific to host" column indicates the host(s) to which each gene was assigned based on our z-score criteria. For each gene, average $\log_2$(CPM+1) expression across hosts, transcript heritability (H^2), Shannon entropy, and functional annotations are provided.

Dataset S11: Information for the 72 isolates of *Botrytis cinerea*. The name of the isolates, geographical origin with latitude and longitude coordinates (when available), the name and affiliation of the person who collected the isolates, the year of isolation and plant species (host) on which the isolate was collected are provided.

Dataset S12: List of reference genomes used for each host species in this study.

Dataset S13: Gene wise negative binomial modeling results for *B. cinerea* transcripts across host species. For each gene, Type II Wald chi square statistics, degrees of freedom, and associated p values are reported for the fixed effects of species, isolate, and their interaction from gene wise negative binomial generalized linear mixed models. The column "variance" represents the proportion of modeled variance attributed to each fixed effect, derived from the conditional variance covariance matrix. The intercept row reflects baseline variance contribution. Models were fitted using a negative binomial distribution with a log link, including tray and sequencing batch as random effects.

Dataset S14. Estimated marginal means of *B. cinerea* gene expression across isolates and host species. The table reports estimated marginal means of *B. cinerea* transcript abundance across 72 *B. cinerea* isolates infecting 10 eudicot host species. Values were derived from a negative binomial GLMM fitted using glmmTMB with a log link function. Columns are defined as follows: **a)** isolate: *B. cinerea* isolate identifier. **b)** species: Host species infected by the isolate. Host codes are Bos, cucumber; Ls, lettuce; Ca, pepper; Ha, sunflower; IT, cowpea; Ucc, bean; Sl, tomato; So, spinach; Bv, chard; Ze, squash. **c)** emmean: Estimated marginal mean of gene expression from the fitted model. **d)** SE: Standard error of the estimated marginal mean. **e)** df: Degrees of freedom associated with the estimate. **f)** asymp.LCL: Asymptotic lower confidence limit of the estimated marginal mean. **g)** asymp.UCL: Asymptotic upper confidence limit of the estimated marginal mean. **h)** gene: Gene identifier corresponding to the *B. cinerea* genome annotation.

References

1. N. E. Soltis, *et al.*, Interactions of tomato and Botrytis cinerea genetic diversity: Parsing the contributions of host differentiation, domestication, and pathogen variation. *Plant Cell* (2019). https://doi.org/10.1105/tpc.18.00857.

2. C. Caseys, *et al.*, Quantitative interactions: The disease outcome of Botrytis cinerea across the plant kingdom. *G3: Genes, Genomes, Genetics* [Preprint] (2021).

3. W. Zhang, *et al.*, Plastic transcriptomes stabilize immunity to pathogen diversity: The jasmonic acid and salicylic acid networks within the Arabidopsis/Botrytis pathosystem open. *Plant Cell* (2017). https://doi.org/10.1105/tpc.17.00348.

4. W. Zhang, *et al.*, Plant–necrotroph co-transcriptome networks illuminate a metabolic battlefield. *Elife* (2019). https://doi.org/10.7554/eLife.44279.

5. S. Atwell, *et al.*, Resequencing and association mapping of the generalist pathogen Botrytis cinerea. *bioRxiv* (2018).

6. J. A. Corwin, *et al.*, The Quantitative Basis of the Arabidopsis Innate Immune System to Endemic Pathogens Depends on Pathogen Genetics. *PLoS Genet.* (2016). https://doi.org/10.1371/journal.pgen.1005789.

7. K. J. Denby, P. Kumar, D. J. Kliebenstein, Identification of Botrytis cinerea susceptibility loci in Arabidopsis thaliana. *Plant Journal* (2004). https://doi.org/10.1111/j.0960-7412.2004.02059.x.

8. T. Mengiste, X. Chen, J. Salmeron, R. Dietrich, The Botrytis Susceptible1 Gene Encodes an R2R3MYB Transcription Factor Protein That Is Required for Biotic and Abiotic Stress Responses in Arabidopsis. *Plant Cell* (2003). https://doi.org/10.1105/tpc.014167.

9. K. Kirkby, S. Roser, K. Plett, Using Detached Industrial Hemp Leaf Inoculation Assays to Screen for Varietal Susceptibility and Product Efficacy on Botrytis cinerea. *Plants* (2023). https://doi.org/10.3390/plants12183278.

10. N. E. Soltis, *et al.*, Pathogen genetic control of transcriptome variation in the arabidopsis thaliana - Botrytis cinerea pathosystem. *Genetics* (2020). https://doi.org/10.1534/genetics.120.303070.

11. C. A. Clark, Comparative Nutrient Dependency of Botrytis squamosa and B. cinerea for Germination of Conidia and Pathogenicity on Onion Leaves. *Phytopathology* (1977). https://doi.org/10.1094/phyto-67-212.

12. J. P. Blakeman, Germination of Botrytis cinerea conidia in vitro in relation to nutrient conditions on leaf surfaces. *Transactions of the British Mycological Society* (1975). https://doi.org/10.1016/s0007-1536(75)80006-4.

13. E. P. Benito, A. Ten Have, J. W. Van ’T Klooster, J. A. L. Van Kan, Fungal and plant gene expression during synchronized infection of tomato leaves by Botrytis cinerea. *Eur. J. Plant Pathol.* (1998). https://doi.org/10.1023/A:1008698116106.

14. R. F. Fordyce, *et al.*, Digital imaging combined with genome-wide association mapping links loci to plant-pathogen interaction traits. *Plant Physiol.* (2018). https://doi.org/10.1104/pp.18.00851.

15. J. Möhring, H. -P. Piepho, Comparison of Weighting in Two-Stage Analysis of Plant Breeding Trials. *Crop Sci.* **49**, 1977–1988 (2009).

16. J. B. Endelman, Fully efficient, two-stage analysis of multi-environment trials with directional dominance and multi-trait genomic selection. *Theoretical and Applied Genetics* **136**, 65 (2023).

17. J. B. Holland, H.-P. Piepho, Don't BLUP Twice. *G3: Genes, Genomes, Genetics* (2024). https://doi.org/10.1093/g3journal/jkae250.

18. D. Bates, M. Mächler, B. M. Bolker, S. C. Walker, Fitting linear mixed-effects models using lme4. *J. Stat. Softw.* (2015). https://doi.org/10.18637/jss.v067.i01.

19. R. Lenth, H. Singman, J. Love, P. Buerkner, M. Herve, Emmeans package: Estimated Marginal means, aka Least-Squares Means. *R package version 1.15-15* [Preprint] (2019).

20. M. W. Fagerland, t-tests, non-parametric tests, and large studies—a paradox of statistical practice? *BMC Med. Res. Methodol.* **12**, 78 (2012).

21. R. Kolde, pheatmap: Pretty Heatmaps version 1.0.12 from CRAN. *https://CRAN.R-project.org/package=pheatmap* (2019).

22. R. Suzuki, Y. Terada, H. Shimodaira, pvclust: Hierarchical Clustering with P-Values via Multiscale Bootstrap Resampling. *R package version 2.2-0* (2019).

23. C. Caseys, D. J. Kliebenstein, Polygenic strategies for host-specific and general virulence of Botrytis cinerea across diverse eudicot hosts. *Genetics* **230**, 79 (2025).

24. R. Kumar, *et al.*, A high-throughput method for Illumina RNA-Seq library preparation. *Front. Plant Sci.* (2012). https://doi.org/10.3389/fpls.2012.00202.

25. P. Ewels, M. Magnusson, S. Lundin, M. Käller, MultiQC: Summarize analysis results for multiple tools and samples in a single report. *Bioinformatics* (2016). https://doi.org/10.1093/bioinformatics/btw354.

26. A. M. Bolger, M. Lohse, B. Usadel, Trimmomatic: A flexible trimmer for Illumina sequence data. *Bioinformatics* (2014). https://doi.org/10.1093/bioinformatics/btu170.

27. D. Kim, J. M. Paggi, C. Park, C. Bennett, S. L. Salzberg, Graph-based genome alignment and genotyping with HISAT2 and HISAT-genotype. *Nat. Biotechnol.* (2019). https://doi.org/10.1038/s41587-019-0201-4.

28. J. A. L. Van Kan, *et al.*, A gapless genome sequence of the fungus Botrytis cinerea. *Mol. Plant Pathol.* (2017). https://doi.org/10.1111/mpp.12384.

29. H. Li, *et al.*, The Sequence Alignment/Map format and SAMtools. *Bioinformatics* (2009). https://doi.org/10.1093/bioinformatics/btp352.

30. M. D. Robinson, D. J. McCarthy, G. K. Smyth, edgeR: A Bioconductor package for differential expression analysis of digital gene expression data. *Bioinformatics* (2009). https://doi.org/10.1093/bioinformatics/btp616.

31. P. Krishnan, *et al.*, Polygenic pathogen networks influence transcriptional plasticity in the Arabidopsis-Botrytis pathosystem. *Genetics* (2023). https://doi.org/10.1093/genetics/iyad099.

32. N. E. Hamilton, M. Ferry, Ggtern: Ternary diagrams using ggplot2. *J. Stat. Softw.* (2018). https://doi.org/10.18637/jss.v087.c03.

33. R. V. Lenth, emmeans: Estimated Marginal Means, aka Least-Squares Means. *CRAN: Contributed Packages* [Preprint] (2017).

34. R. Lenth, H. Singman, J. Love, P. Buerkner, M. Herve, Emmeans package: Estimated Marginal means, aka Least-Squares Means. *R package version 1.15-15* [Preprint] (2019).

35. A. J. Ameri, Z. A. Lewis, Shannon entropy as a metric for conditional gene expression in Neurospora crassa. *G3: Genes, Genomes, Genetics* (2021). https://doi.org/10.1093/g3journal/jkab055.

36. N. D. Heintzman, *et al.*, Histone modifications at human enhancers reflect global cell-type-specific gene expression. *Nature* (2009). https://doi.org/10.1038/nature07829.

37. X. Zhang, *et al.*, Genome-wide High-Resolution Mapping and Functional Analysis of DNA Methylation in Arabidopsis. *Cell* (2006). https://doi.org/10.1016/j.cell.2006.08.003.

38. G. F. Jenks, The data model concept in statistical mapping. *International yearbook of cartography* (1967).

39. M. M. Mukaka, Statistics corner: A guide to appropriate use of correlation coefficient in medical research. *Malawi Medical Journal* (2012).

40. P. Lu, *et al.*, Genome-Wide Identification of Alternative Splicing in Botrytis cinerea During Infection Stage of Solanum lycopersicum. *Microorganisms* **13**, 360 (2025).

41. Y. You, *et al.*, Botrytis cinerea combines four molecular strategies to tolerate membrane-permeating plant compounds and to increase virulence. *Nature Communications 2024 15:1* **15**, 1–17 (2024).

42. S. Atwell, *et al.*, Whole genome resequencing of Botrytis cinerea isolates identifies high levels of standing diversity. *Front. Microbiol.* (2015). https://doi.org/10.3389/fmicb.2015.00996.

43. J. A. L. Van Kan, *et al.*, A gapless genome sequence of the fungus Botrytis cinerea. *Mol. Plant Pathol.* **18**, 75–89 (2016).

44. H. Li, Aligning sequence reads, clone sequences and assembly contigs with BWA-MEM. *arxiv.orgH LiarXiv preprint arXiv:1303.3997, 2013•arxiv.org* (2013).

45. E. Garrison, G. Marth, "Haplotype-based variant detection from short-read sequencing" (2012).

46. C. W. Nelson, L. H. Moncla, A. L. Hughes, SNPGenie: Estimating evolutionary parameters to detect natural selection using pooled next-generation sequencing data. *Bioinformatics* (2015). https://doi.org/10.1093/bioinformatics/btv449.
